# Rare, Tightly-Bound, Multi-Cellular Clusters in the Pancreatic Ducts of Adult Mice Function Like Progenitor Cells and Survive and Proliferate After Acinar Cell Injury

**DOI:** 10.1093/stmcls/sxae005

**Published:** 2024-01-11

**Authors:** Jacob R Tremblay, Jose A Ortiz, Janine C Quijano, Heather N Zook, Neslihan Erdem, Jeanne M LeBon, Wendong Li, Kevin Jou, Walter Tsark, Jeffrey R Mann, Mark T Kozlowski, David A Tirrell, Farzad Esni, Dannielle D Engle, Arthur D Riggs, Hsun Teresa Ku

**Affiliations:** Department of Translational Research and Cellular Therapeutics, Arthur Riggs Diabetes and Metabolism Research Institute, Beckman Research Institute, City of Hope, Duarte, CA, USA; The Irell and Manella Graduate School of Biological Sciences, Beckman Research Institute, City of Hope, Duarte, CA, USA; Department of Translational Research and Cellular Therapeutics, Arthur Riggs Diabetes and Metabolism Research Institute, Beckman Research Institute, City of Hope, Duarte, CA, USA; The Irell and Manella Graduate School of Biological Sciences, Beckman Research Institute, City of Hope, Duarte, CA, USA; Department of Translational Research and Cellular Therapeutics, Arthur Riggs Diabetes and Metabolism Research Institute, Beckman Research Institute, City of Hope, Duarte, CA, USA; Department of Translational Research and Cellular Therapeutics, Arthur Riggs Diabetes and Metabolism Research Institute, Beckman Research Institute, City of Hope, Duarte, CA, USA; The Irell and Manella Graduate School of Biological Sciences, Beckman Research Institute, City of Hope, Duarte, CA, USA; Department of Translational Research and Cellular Therapeutics, Arthur Riggs Diabetes and Metabolism Research Institute, Beckman Research Institute, City of Hope, Duarte, CA, USA; The Irell and Manella Graduate School of Biological Sciences, Beckman Research Institute, City of Hope, Duarte, CA, USA; Department of Translational Research and Cellular Therapeutics, Arthur Riggs Diabetes and Metabolism Research Institute, Beckman Research Institute, City of Hope, Duarte, CA, USA; Department of Translational Research and Cellular Therapeutics, Arthur Riggs Diabetes and Metabolism Research Institute, Beckman Research Institute, City of Hope, Duarte, CA, USA; Department of Translational Research and Cellular Therapeutics, Arthur Riggs Diabetes and Metabolism Research Institute, Beckman Research Institute, City of Hope, Duarte, CA, USA; Center for Comparative Medicine, Beckman Research Institute, City of Hope, Duarte, CA, USA; Center for Comparative Medicine, Beckman Research Institute, City of Hope, Duarte, CA, USA; Division of Chemistry and Chemical Engineering, California Institute of Technology, Pasadena, CA, USA; Division of Chemistry and Chemical Engineering, California Institute of Technology, Pasadena, CA, USA; Department of Surgery, University of Pittsburgh School of Medicine, Pittsburgh, PA, USA; Regulatory Biology Laboratory, Salk Institute for Biological Studies, La Jolla, CA, USA; Department of Translational Research and Cellular Therapeutics, Arthur Riggs Diabetes and Metabolism Research Institute, Beckman Research Institute, City of Hope, Duarte, CA, USA; Department of Translational Research and Cellular Therapeutics, Arthur Riggs Diabetes and Metabolism Research Institute, Beckman Research Institute, City of Hope, Duarte, CA, USA; The Irell and Manella Graduate School of Biological Sciences, Beckman Research Institute, City of Hope, Duarte, CA, USA

**Keywords:** pancreas, ductal cell clusters, adult progenitor cells, acinar cell injury, proliferation, diabetes, cancer, Biological sciences, Cell biology

## Abstract

Pancreatic ductal progenitor cells have been proposed to contribute to adult tissue maintenance and regeneration after injury, but the identity of such ductal cells remains elusive. Here, from adult mice, we identify a near homogenous population of ductal progenitor-like clusters, with an average of 8 cells per cluster. They are a rare subpopulation, about 0.1% of the total pancreatic cells, and can be sorted using a fluorescence-activated cell sorter with the CD133^high^CD71^low^FSC^mid-high^ phenotype. They exhibit properties in self-renewal and tri-lineage differentiation (including endocrine-like cells) in a unique 3-dimensional colony assay system. An in vitro lineage tracing experiment, using a novel *Hprt*^*DsRed/+*^ mouse model, demonstrates that a single cell from a cluster clonally gives rise to a colony. Droplet RNAseq analysis demonstrates that these ductal clusters express embryonic multipotent progenitor cell markers Sox9, Pdx1, and Nkx6-1, and genes involved in actin cytoskeleton regulation, inflammation responses, organ development, and cancer. Surprisingly, these ductal clusters resist prolonged trypsin digestion in vitro, preferentially survive in vivo after a severe acinar cell injury and become proliferative within 14 days post-injury. Thus, the ductal clusters are the fundamental units of progenitor-like cells in the adult murine pancreas with implications in diabetes treatment and tumorigenicity.

## Graphical Abstract

**Graphical Abstract:**
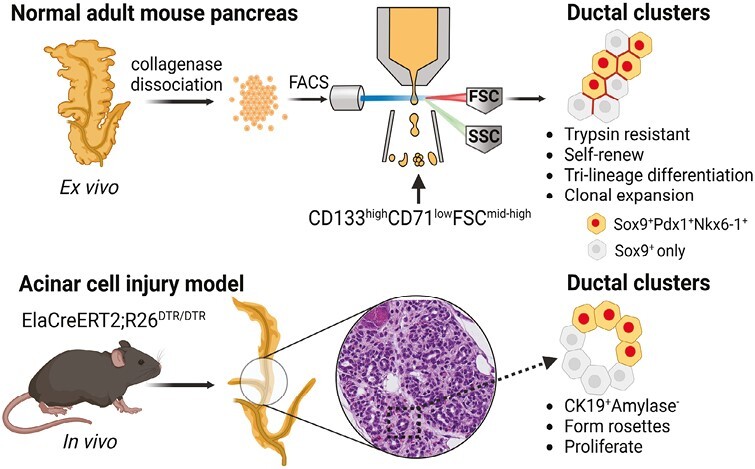

**Significance Statement**

Adult tissue-specific progenitor cells play important roles in homeostasis and regeneration. Here from normal mice, we purify ductal progenitor-like cell clusters from pancreas to near homogeneity. They are rare (~0.1% of total pancreatic cells) but with capacities of self-renewal and tri-lineage differentiation including endocrine-like cells. They express many embryonic multipotent progenitor markers. Surprisingly, these clusters resist trypsin digestion in vitro as well as in vivo after a severe acinar cell injury, and become proliferative. These rare ductal progenitor clusters have implications in diabetes treatment as well as tumorigenicity due to their survival advantage.

**Highlights**

- Rare ductal cell clusters are identified as the units of progenitor cells.
- Ductal clusters can differentiate into multiple lineages and self-renew ex vivo.
- Cells in clusters are tightly bound and resistant to prolonged trypsin digestion.
- Ductal progenitor clusters survive and proliferate after acinar cell injury in mice.

## Introduction

In the adult pancreatic epithelium, there are 3 major lineages of cells: acinar, ductal, and endocrine cells. Acinar and ductal cells (collectively known as the exocrine tissue) are responsible for secreting and transporting digestive enzymes, respectively, to aid in nutrient digestion while endocrine cells secrete hormones to regulate glucose homeostasis. Adult pancreatic cells are mostly quiescent during steady state.^1-3^ However, when damage and stress occur to acinar or endocrine insulin-producing beta cells,^4-7^ which results in pancreatitis or diabetes, respectively, proliferation increases not only in acinar and endocrine cells but also in the ductal cells.^8,9^

Adult ductal cells have been implicated as facultative progenitor cells that contribute to beta cell neogenesis,^10^ but this research topic remains controversial.^11,12^ For example, during homeostasis, pregnancy, or certain injuries, new beta cells are found to be mostly originated from preexisting beta cells, as demonstrated using insulin promoter-driven lineage tracing in adult mice.^13,14^ When a pan-ductal cell marker, such as Hnf1b^15^ or Sox9,^16^ was used for in vivo lineage tracing, the minimal contribution from ductal to beta cells was observed. Together, these results led to the conclusion that adult ductal cells do not contribute to new beta cells. However, these studies assumed adult ductal cell homogeneity. Emerging research demonstrates that adult ductal cells are heterogenous,^17-21^ and a certain subpopulation of adult ductal cells is involved in the regeneration of endocrine cells during insulin resistance^22^ and insulin-dependent diabetes.^23,24^ These in vivo results are also supported by the demonstration of in vitro self-renewal and endocrine differentiation of some ductal cells from adult pancreas, which used fluorescence-activated cell sorting (FACS) to enrich progenitor-like cells from mice^25-28^ or humans.^20,29,30^ We have previously shown that the sorted CD133^high^CD71^low^ ductal cells from normal adult mice are a subpopulation of total ductal cells, and are enriched for progenitor-like cells that both self-renew and give rise to the three main pancreatic lineages, as assayed by a unique, 3D colony assay system permissive for tri-lineage differentiation.^31^ However, purification of these progenitor-like cells has not been achieved.

Here we report purification to near homogeneity of ductal cell clusters that behave like progenitor cells demonstrated through self-renewal and tri-lineage differentiation. These ductal clusters simultaneously express embryonic multipotent progenitor cell (MPC) markers: Sox9, Pdx1, and Nkx6-1.^32,33^ Much to our surprise, these ductal clusters are resistant to dissociation by enzymic digestion in vitro as well as in vivo after acinar cell injury. The fact that these ductal clusters only constitute 0.1% of the total pancreatic cells may explain the difficulties in studying these rare ductal progenitor-like cells in the past.

## Material and Methods

### Mice

Animal experiments were conducted according to the Institutional Animal Care and Use Committee at the City of Hope (protocol #11017). C57BL/6J (B6) mice (The Jackson Laboratory) (both sexes) were used in most experiments unless specified otherwise. Transgenic *ElaCreERT2;R26^DTR/DTR^* mice were used as reported previously^34^ but were crossed to B6 mice to yield > 99.99% B6 genetic background. FVB.129S1-Hprt^tm1(CAG-DsRed)Mnn/COH^ (*Hprt^DsRed/+^*) mice were generated in-house using gene targeting vectors and embryonic stem cell clone selection strategies. All experiments were conducted using mice between 8 and 12 weeks of age.

### Dissociation of Pancreas, Sorting, and Flow Cytometry Analysis

These procedures were performed as previously described.^31^

### Colony Assay

Sorted cells/units were resuspended at a density of 500 cells/units per well per 0.5 mL for the Matrigel/RSPO1 colony assay or 8.0 × 10^3^ cells/units per well per 0.5 mL for the laminin hydrogel assay, as described previously.^35^ Colonies grown in Matrigel/RSPO1 or laminin assay were counted 2–3 weeks or 10 days after plating, respectively.

### Micro-Manipulation of Single Cells/Units or Colonies

Single freshly sorted cells/units or individual colonies were lifted one-by-one as described previously.^36^

### Serial Dissociation and Replating of Colonies

Colonies grown in Matrigel/RSPO1 assay were serially collected, dissociated by collagenase B followed by 0.25% (wt/vol) trypsin-EDTA, and replated into a new Matrigel/RSPO1 colony assay, as described previously.^36^

### Conventional or Microfluidic Quantitative Reverse Transcription-Polymerase Chain Reaction (qRT-PCR).

Conventional and microfluidic qRT-PCR analyses were conducted as reported by Jin et al.^35^ The internal control was beta-actin. All experiments were performed with negative (water) and positive controls (adult B6 pancreatic cells). Taqman probes used are listed in Supplementary Table S1.

### Droplet-Based RNA-Sequencing and Data Analysis

CD133^high^CD71^low^FSC^mid-high^ and CD133^high^CD71^low^FSC^low^ fractions were sorted and subjected to a 10x Chromium device using a 10X V3 Single Cell 3ʹ Solution kit (10x Genomics, Chromium Single Cell 3ʹ Regent 00kit V3 Chemistry, Cat. PN-1000092). Approximately 0.1 million reads per cell/unit were sequenced. Raw sequencing data were aligned to the mouse genome (mm10) and the R package Seurat was used for data analysis. Units with <200 detectable genes and >15% mitochondrial genes were excluded. Raw data had been deposited into the Gene Expression Omnibus (GEO) database, code GSE249084.

### Immunostaining of Small Clusters and Colonies

Small clusters or colonies were fixed in 4% paraformaldehyde (PFA) containing 0.15% Triton X-100 at 4 °C overnight, washed with phosphate buffered saline, cryoprotected at 4 °C overnight in 30% sucrose, followed by frozen embedding in OCT compound (ThermoFisher). Frozen blocks were sectioned (8 μm thickness) onto glass slides (Fisher Scientific) before staining. For whole-mount staining, colonies or control tissues (ie, exocrine or islets) were fixed in 4% PFA containing 0.15% Triton X-100 for 1 hour, followed by immunostaining. Antibodies used are listed in Supplementary Table S2.

### Histology and Staining in Pancreas Tissue

Pancreata were dissected, fixed in 10% formalin solution, paraffin embedded, sectioned (5 μm thickness) onto glass slides, and stained with antibodies. For EdU detection, Click-iT EdU Alexa Fluor 555 Imaging Kit (Thermo Fisher Scientific) was used following the manufacturer’s instructions. Antibodies used are listed in Supplementary Table S2.

### Acinar Cell Injury in Mice

Tamoxifen (TAM) was injected intraperitoneally into *ElaCreERT2;R26^DTR/DTR^* or control mice at 0.2 mg/g body weight (b.w.) once per day, every other day, for a total of three injections. Three weeks later, a high dose of diphtheria toxin (DT) (200 ng/20g b.w.) was injected once per day for 3 days. For EdU labeling, control and injured adult mice were injected with EdU (100 mg/kg b.w., Abcam) every 24 hours for 3 days prior to the procurement of the pancreas. Mice were euthanized 3 or 14 days after the last DT injection.

### Proliferation Analysis

Representative pancreatic sections (100 μm apart) were subjected to staining described above and images were taken at 20× magnification with identical channel exposure time for control and injured samples. Images were processed using QuPath v0.2.3 software.^37^

### Statistical Analysis

GraphPad Prism 8 software was used for statistical analysis. Data format, presented as mean ± SD or mean ± SEM, and sample size are indicated in the figure legend. Significance is defined as *P < .05, **P < .01, ***P < .001, ****P < .0001.

Additional experimental methods and details are provided in the Supplemental information.

## Results

### The Ductal CD133^high^CD71^low^FSC^mid-high^ Fraction Exhibits “Small Cluster” Morphology and Is Highly Enriched for Pancreatic Colony-Forming Units (PCFUs)

**Figure 1.**
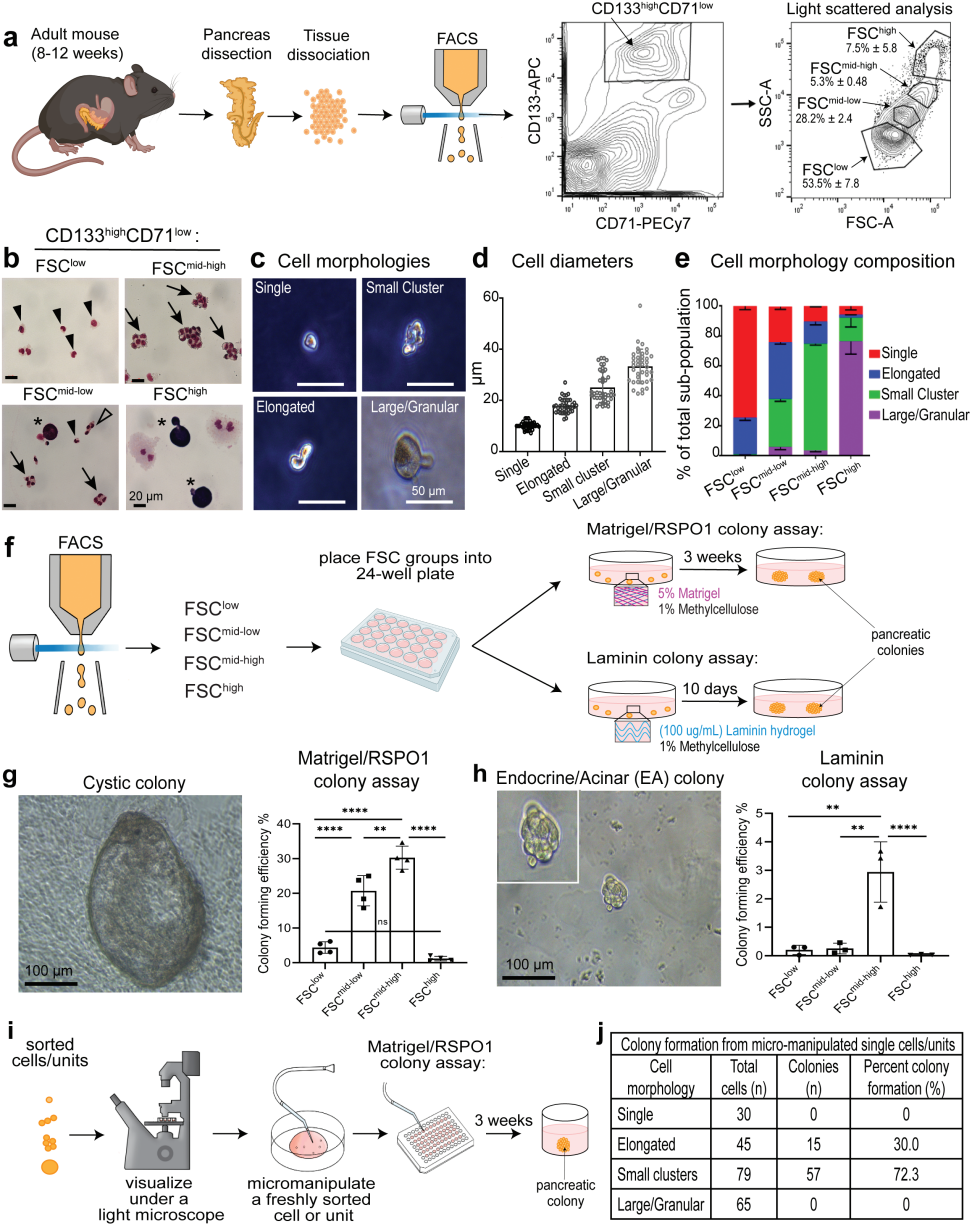
The ductal CD133^high^CD71^low^FSC^mid-high^ fraction is enriched for cells with “small cluster” morphology and is highly enriched for colony-forming units. (**a**) Schematic of experimental design with representative flow cytometry analysis. Note that a CD133^high^CD71^low^FSC fraction is abbreviated as “FSC.” (**b**) Representative photomicrographs of sorted fractions stained with Wright-Giemsa solution. Arrow heads, Single; Unfilled arrow heads, Elongated; arrows, Small cluster; stars, Large/Granular morphology. Scale bar = 20 µm. (**c**) Representative photomicrographs of cell morphologies observed under a phase-contrast light microscope. Scale bar = 50 µm. (**d**) Measurement of diameters revealed that different morphologies had sizes consistent with their forward scatter in flow cytometry analysis. *n* = 2 independent experiments from 5 mice each and at least 20 replicates in each group. Error bars represent SD. (**e**) Frequencies of each morphology in each sorted subpopulation. Small clusters are most enriched in the FSC^mid-high^ fraction. *n* = 3 independent sorted experiments for each gate, with 3 different fields of view when counting cell types. Error bars represent SEM. (**f**) Schematic of experimental design with sorted units placed into either the Matrigel/RSPO1 colony assay (contains 5% Matrigel and 1% methylcellulose) or the Laminin colony assay (contains 100 µg/mL laminin hydrogel and 1% methylcellulose), followed by incubation to produce colonies for subsequent analyses. (**g**) Representative image of a 3-week-old Cystic colony grown in Matrigel/RSPO1 colony assay (left). Colony-forming efficiency was determined for each FSC fraction (right). *n* = 5 independent experiments. (h) Representative image of an endocrine/acinar (E/A) colony grown in Laminin colony assay (left) and colony-forming efficiency (right). *n* = 3 independent experiments. Statistics were performed using one-way ANOVA multiple comparisons. Error bars represent SEM. (**i–j**) Schematic of micro-manipulation of various morphologies and plating into Matrigel/RSPO1 colony assay to determine percent colony formation. Among the 79 micro-manipulated small clusters, 72.3% gave rise to colonies, demonstrating a near homogeneous population of progenitor-like cells.

In flow cytometry, the light scatters in line with the laser beam, called forward scatter (FSC), are indicative of particle size, whereas those in perpendicular are called side scatter (SSC), which reflects complexity of the particles that pass through the beam. FSC and SSC have been useful to distinguish various cell types.^38^ Owing to a general quiescent state of the adult pancreatic cells, we hypothesized that adult pancreatic progenitor cells may be small in size with a high nuclear-to-cytoplasm ratio, similar to adult hematopoietic stem cells.^39,40^ To test this hypothesis, we fractioned according to FSC and SSC parameters on the ductal CD133^high^CD71^low^ population, which was previously identified as enriched for progenitor-like cells.^31^ Four fractions were identified among the parent CD133^high^CD71^low^ population, which we called FSC^low^, FSC^mid-low^, FSC^mid-high^, and FSC^high^ (Fig. 1a, Supplementary Fig. S1); these 4 fractions constituted 1.27 ± 0.27, 0.47 ± 0.13, 0.12 ± 0.04, and 0.23 ± 0.16% of the total pancreatic cells, and 53.5 ± 7.8, 28.2 ± 2.4, 5.3 ± 0.5, and 7.5 ± 5.8% of the gated CD133^high^CD71^low^ population, respectively.

Next, we examine the cell morphology of freshly sorted fractions with Wright-Giemsa staining. The FSC^low^ fraction (Fig. 1b, upper left panel) was comprised of mostly single cells, which we named “single.” The FSC^mid-low^ fraction (Fig. 1b, lower left) contained a mixture of different cell morphologies, including the “elongated” morphology. The FSC^mid-high^ fraction (Fig 1b, upper right) contained aggregates of cells that were highly resistant to trypsin dissociation, which we named “small clusters.” The “large/granular” morphology in the FSC^high^ fraction (Fig. 1b, lower right) had 2 morphologies: bi-nucleated cells with pink cytoplasm suggestive of acinar cells,^41^ and single-nucleated cells with purple cytoplasm; the identity of which is currently unknown. These 4 morphologies were also distinguishable under a phase-contrast light microscope (Fig. 1c). The diameters of these morphologies were measured; “single” had the smallest diameters, while “elongated,” “small clusters,” and “large/granular” had increasingly larger diameters (Fig. 1d). Counting the 4 morphologies in each sorted fraction revealed that higher FSC was positively correlated with an increased proportion of morphologies with larger diameters (Fig. 1e), confirming effective sorting based on FSC.

To determine which fraction contains progenitor-like cells, we employed our pancreatic colony assay system using methylcellulose as a matrix.^35^ We named a progenitor cell capable of giving rise to a pancreatic colony a “pancreatic colony-forming unit (PCFU).” Freshly sorted fractions were plated into colony assays and the resulting colonies analyzed (Fig. 1f). In the colony assay containing Matrigel (5% vol/vol) and Rspondin-1 (RSPO1)^35^ (herein Matrigel/RSPO1 colony assay), the FSC^mid-high^ fraction gave rise to the highest number of “Cystic” colonies, compared to other fractions (Fig. 1g). The FSC^low^ fraction also gave rise to some cystic colonies, but the colony-forming efficiency was approximately 7-fold lower than the FSC^mid-high^ fraction (Fig. 1g). Next, in the colony assay containing an artificial ECM protein fitted with laminin IKVAV-containing sequence (herein laminin colony assay), and in the absence of Matrigel and RSPO1,^35,42^ the FSC^mid-high^ fraction also gave rise to the highest number of colonies (named “endocrine/acinar [E/A]” colonies) (Fig. 1h), with an approximately 250-fold higher colony-forming efficiency compared to the FSC^low^ fraction.

To determine which cell morphology was responsible for forming colonies, different morphologies were micro-manipulated one-by-one and placed into Matrigel/RSPO1 colony assay at 1 cell (or cluster) per well in 96-well plates (Fig. 1i). The single and large morphologies did not give rise to colonies, whereas elongated and small clusters displayed 30% and 72.3% colony-forming efficiency, respectively (Fig. 1j). These results demonstrate that the small clusters are the predominant contributors to colony formation. Because the FSC^mid-high^ fraction is most enriched for PCFUs in both assays, we focused our attention on this fraction for subsequent analyses.

### The Ductal CD133^high^CD71^low^FSC^mid-high^ Fraction is Capable of Tri-Lineage Differentiation I*n* Vitro

**Figure 2.**
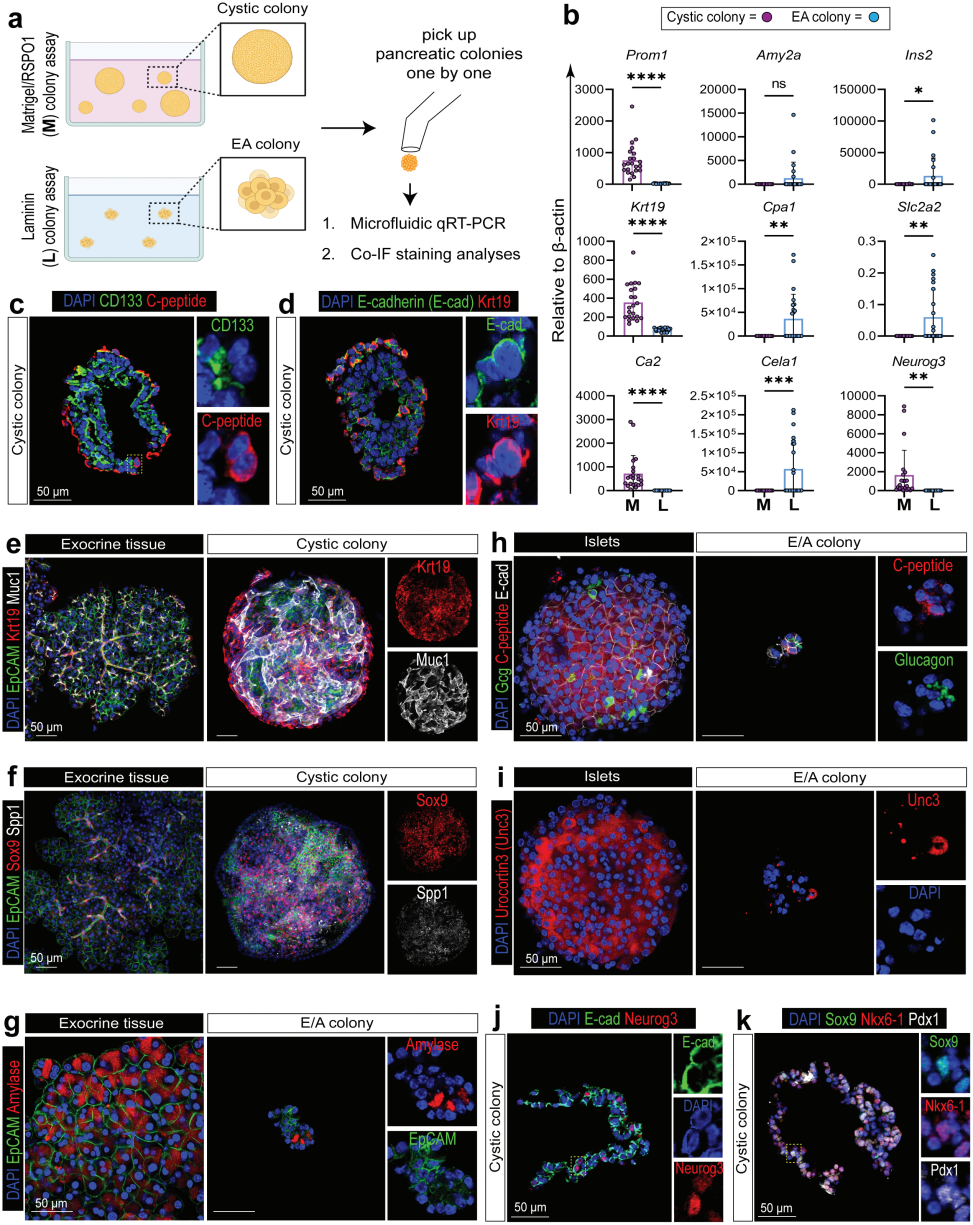
The small clusters are tri-potent capable of giving rise to ductal, acinar, and endocrine like cells in colonies. (**a**) Schematic of experimental design. (**b**) Microfluidic qRT-PCR analysis shows that individual FSC^mid-high^-derived colonies grown in Matrigel (M) preferentially express ductal (*Prom1, Krt19, Ca2*) and endocrine progenitor cell markers (*Neurog3*), while those grown in laminin (L) preferentially express acinar (*Amy2a, Cpa1, Cela1*) or endocrine markers (*Ins2, Slc2a2*), consistent with our prior finding.^30^ Each dot represents a colony. *n* = 22-23. Statistics were performed comparing cystic vs E/A colonies using 2-tailed Student’s *t*-test. Error bars represent SD. (**c–d**) Immunofluorescence (IF) staining of Cystic colonies in frozen sections with epithelial (E-cadherin), ductal (CD133, Krt19), and endocrine (C-peptide) markers. (**e-i**) Whole-mount IF staining with ductal (Krt19, Muc1, Sox9, Spp1) in cystic colonies, as well as acinar (amylase) and endocrine markers (C-peptide, glucagon, Urocortin 3) in E/A colonies. EpCAM and E-cad are epithelial cell markers. Exocrine or islet tissues served as positive controls. (**j–k**) IF staining of Cystic colonies in frozen sections with endocrine progenitor (Neurog3) and pancreatic progenitor cell markers (Sox9, Nkx6-1, Pdx1). Dashed boxes are enlarged on the right. Scale bars = 50 µm.

The lineage potential of a PCFU is reflected in the lineage composition of the colony to which the PCFU gives rise. To determine lineage marker expression, individual cystic or E/A colonies were micro-manipulated (handpicked one-by-one) and analyzed for gene expression by microfluidic qRT-PCR analysis or protein expression by immunofluorescence (IF) analyses (Fig. 2a). Consistent with our prior findings,^35,42^ cystic colonies expressed higher levels of markers for ductal (*Prom1, Krt19, Ca2*) and endocrine progenitors (*Neurog3*) when grown in the Matrigel/RSPO1, compared to the E/A colonies grown in the laminin colony assay. In contrast, E/A colonies expressed higher levels of acinar (*Cpa1, Cela1*) and endocrine markers (*Ins2, Slc2a2*), compared to Cystic colonies (Fig. 2b). Protein expression of CD133, C-peptide (Fig. 2c), Krt19 (a.k.a. CK19), E-cadherin, Muc1 (Fig. 2d–2e, Supplementary Fig. S2a), Sox9, and Spp1 (Fig. 2; Supplementary Fig. S2b) was confirmed in the Cystic colonies. Amylase, EpCAM (Fig. 2g; Supplementary Movie S1; Supplementary Fig. S2c), C-peptide, glucagon (Fig. 2h, Supplementary Movie S2 and Fig. S2d), and Urocortin3 (Fig. 2i, Supplementary Fig. S2e) were confirmed in E/A colonies. We also confirmed in Cystic colonies protein expression of Neurog3 (Fig. 2j, Supplementary Fig. S2f). Together, these results demonstrate that PCFUs in the FSC^mid-high^ fraction are tri-potent for the 3 major pancreatic lineage cells in vitro.

### The Ductal CD133^high^CD71^low^FSC^mid-high^ Fraction Self-Renews Robustly In Vitro

**Figure 3.**
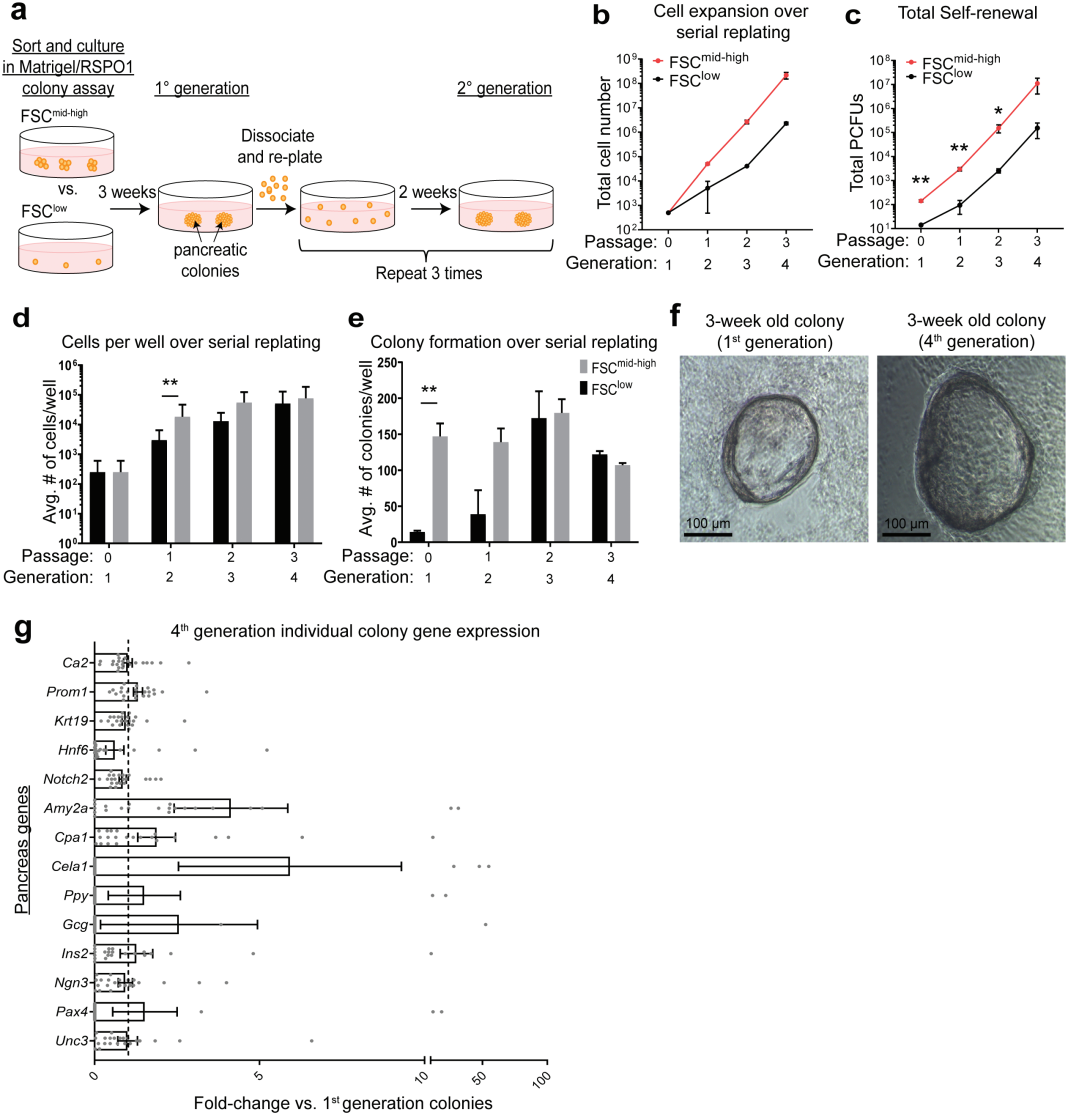
The ductal CD133^high^CD71^low^FSC^mid-high^ fraction self-renews robustly in vitro. (**a**) Schematic of serial replating strategy to assess self-renewal capacity. (**b–c**) Cell growth curves from the CD133^high^CD71^low^FSC^low^ (FSC^low^) or the CD133^high^CD71^low^FSC^mid-high^ (FSC^mid-high^) fraction over time. *n* = 2 (b) or 3 (c) independent experiments consisting of 4 technical replicates each. Error bars represent SEM. (**d**) Average number of cells per well over serial replating. (**e**) Average number of colonies per well over serial replating. (**f**) Photomicrographs of representative 3-week-old Cystic colonies from the 1st and the 4th generations. (**g**) Individual colonies were micro-manipulated and gene expression analyzed using microfluidic qRT-PCR for ductal (*Ca2, Prom1, Krt19*), acinar (*Amy2a, Cpa1, Cela1*), endocrine (*Ppy, Gcg, Ins2, Ngn3*), and other markers (*Hnf6, Notch2, Pax4, Unc3*). Data represent fold-change calculation of gene expression in colonies of the 4th generation compared to the 1st generation. Each data point is a colony. *n* = 22 individual colonies from each group. Error bars represent SEM. Statistics were performed comparing FSC^mid-high^ vs. FSC^low^ using two-tailed Student’s *t*-test.

As mentioned, murine embryonic MPCs co-express Sox9, Nkx6-1, and Pdx1.^32,33^ We found that some cells in the adult cystic colonies were triple-positive (TP) for Sox9, Nkx6-1, and Pdx1 (Fig. 2k, Supplementary Fig. S2g), which prompted us to examine the self-renewal potential of the FSC^mid-high^ fraction, by using a serial dissociation and replating strategy.^35^ The FSC^mid-high^ or the FSC^low^ fractions were plated into the Matrigel/RSPO1 colony assay, and the resulting 3-week-old primary colonies were dissociated and serially replated for a total of 4 generations (Fig. 3a). The FSC^mid-high^ fraction grew exponentially from the first generation and gave rise to a higher number of total cells (PCFUs plus non-PCFUs) (Fig. 3b) as well as total PCFUs (Fig. 3c), compared to the FSC^low^ fraction. Although the FSC^low^ fraction had a lag phase in the early passages, the growth rate (slope of the curve) caught up with the FSC^mid-high^ fraction in the later passages (Fig. 3b–3c), in both the proportion of the total cells (Fig. 3d) and total colonies per well (Fig. 3e). Cells with the elongated morphology in the sorted FSC^low^ fraction were the likely source of Cystic colonies that self-renewed (Fig. 1e and 1j); however, we cannot rule out the possibility that a few small clusters were present in the FSC^low^ fraction. After 9 weeks in the self-renewing culture condition, the total cell number and total PCFUs from the FSC^mid-high^ fraction expanded ~440,000 and ~78,000 fold, respectively (Fig. 3b, 3c). We confirmed that there was no observable difference in the Cystic colony morphology (Fig. 3f) or gene expression (Fig. 3g) between the 1st and 4th generation, demonstrating that PCFUs were maintained over multiple generations in vitro. Together, these results demonstrate a robust self-renewal ability of the small clusters.

### Cell-Tracing Analysis Reveals Only One Cell in a Ductal Small Cluster Gives Rise to a Cystic Colony

To determine whether a single cell within a small cluster was sufficient to form a colony, we generated *Hprt*^*DsRed/+*^ mice in which the gene for *Disocosoma* sp. red fluorescent protein (*DsRed*) replaced hypoxanthine guanine phosphoribosyl transferase (*Hprt*) on one X chromosome. This allowed DsRed to report random X inactivation in the pre-implantation female embryos.^43^ Because X inactivation is somatically heritable with extreme fidelity,^44^ the progeny of a DsRed^+^ cell will always be red and the progeny of a DsRed^−^ cell will always be devoid of the fluorescence signal. Thus, heterozygous female *Hprt*^*DsRed/+*^ mice were expected to display mosaic pattern with half DsRed^+^ and half DsRed^−^ cells, which was confirmed by IF staining of endogenous pancreas and flow cytometry analysis of splenocytes (Supplementary Fig. S3).

**Figure 4.**
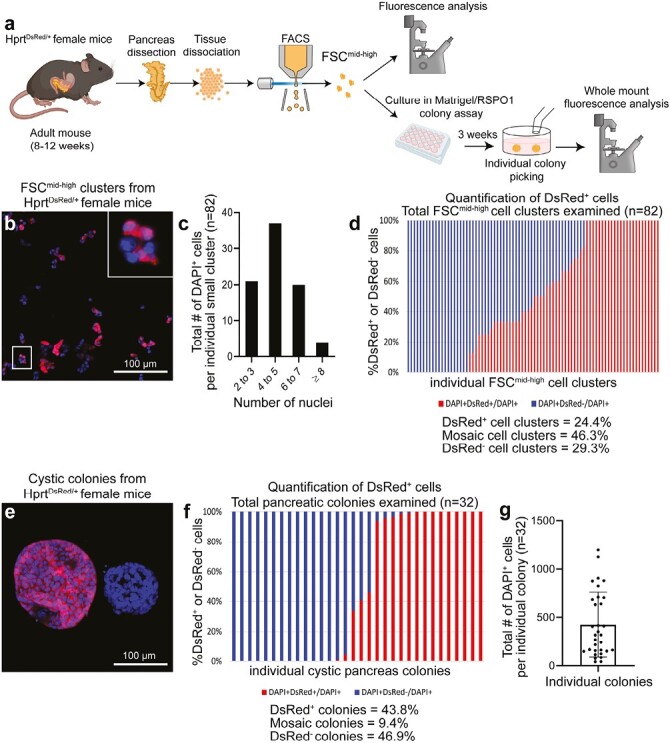
Cell-tracing using a random X inactivation strategy reveals that only one cell in a ductal small cluster gives rise to a colony. (**a**) Schematic of experimental design. (**b**) Representative images of small clusters from the CD133^high^CD71^low^FSC^mid-high^ (FSC^mid-high^) fraction of *Hprt^DsRed/+^* heterozygous female mice. Nuclei were stained with DAPI. The zoomed-in image shows a mosaic small cluster with a mixture of DsRed^+^ (red) and DsRed^−^ cells. Scale bar = 100 µm. (**c**) Distribution of the number of nuclei per small cluster. *n* = 82 clusters. (**d**) The proportion of DsRed^+^ and DsRed^−^ cells was determined in each cluster. Each bar represents a small cluster, *n* = 82. The clusters were categorized as DsRed^+^ (defined as > 90% red cells; 24.4% among all clusters examined), mosaic (46.3%), or DsRed^−^ (defined as < 10% red; 29.3%). (**e**) The FSC^mid-high^ fraction from *Hprt^DsRed/+^* female mice were plated into Matrigel/RSPO1 colony assay. Three weeks later, colonies were collected and imaged on a confocal microscope from top to bottom of each colony. Two representative Cystic colonies are shown in maximum intensity projection of all image slices. Scale bar = 100 µm. (**f**) Colonies were categorized as DsRed^+^ (defined as > 90% red; 43.8% among all colonies examined), mosaic (9.4%), or DsRed^−^ (defined as < 10% red; 46.9%). Each bar represents a colony, *n* = 32. (**g**) The number of DAPI^+^ cells from each colony was quantified.

We next analyzed the ductal small clusters from heterozygous female *Hprt*^*DsRed/+*^ mice (Fig. 4a). Half of the freshly sorted small clusters were mosaic (containing both DsRed^+^ and DsRed^−^ cells, 46.3%), while a quarter of small clusters were either fully labeled (DsRed^+^, 24.4%) or unlabeled (DsRed^−^, 29.3%; Fig. 4b–4d), which is consistent with random X inactivation.

Next, small clusters from heterozygous female *Hprt*^*DsRed/+*^ mice were plated into Matrigel/RSPO1 colony assay and cultured for 3 weeks (Fig. 4a, lower). Individual colonies were examined under a confocal microscope using optical slice z-stacks (Fig. 4e). About half of the colonies were either fully labeled (43.8%, DsRed^+^) or unlabeled (46.9%, DsRed^−^), while only a few colonies were mosaic (9.4%, Fig. 4f), demonstrating a change of mosaics in the population of colonies compared to their ancestors. These results demonstrate the clonal expansion of a single cell among the multi-cellular ductal cluster to form a colony. Interestingly, the number of cells in individual colonies were not uniform (Fig. 4g), suggesting variable proliferative potentials of the originating progenitor cells.

### The Ductal Small Clusters is Enriched for Genes Involved in Cell–Cell, Cell–Matrix Interactions, Organ Development, Response to Wounding, and Pancreatic Cancer

**Figure 5.**
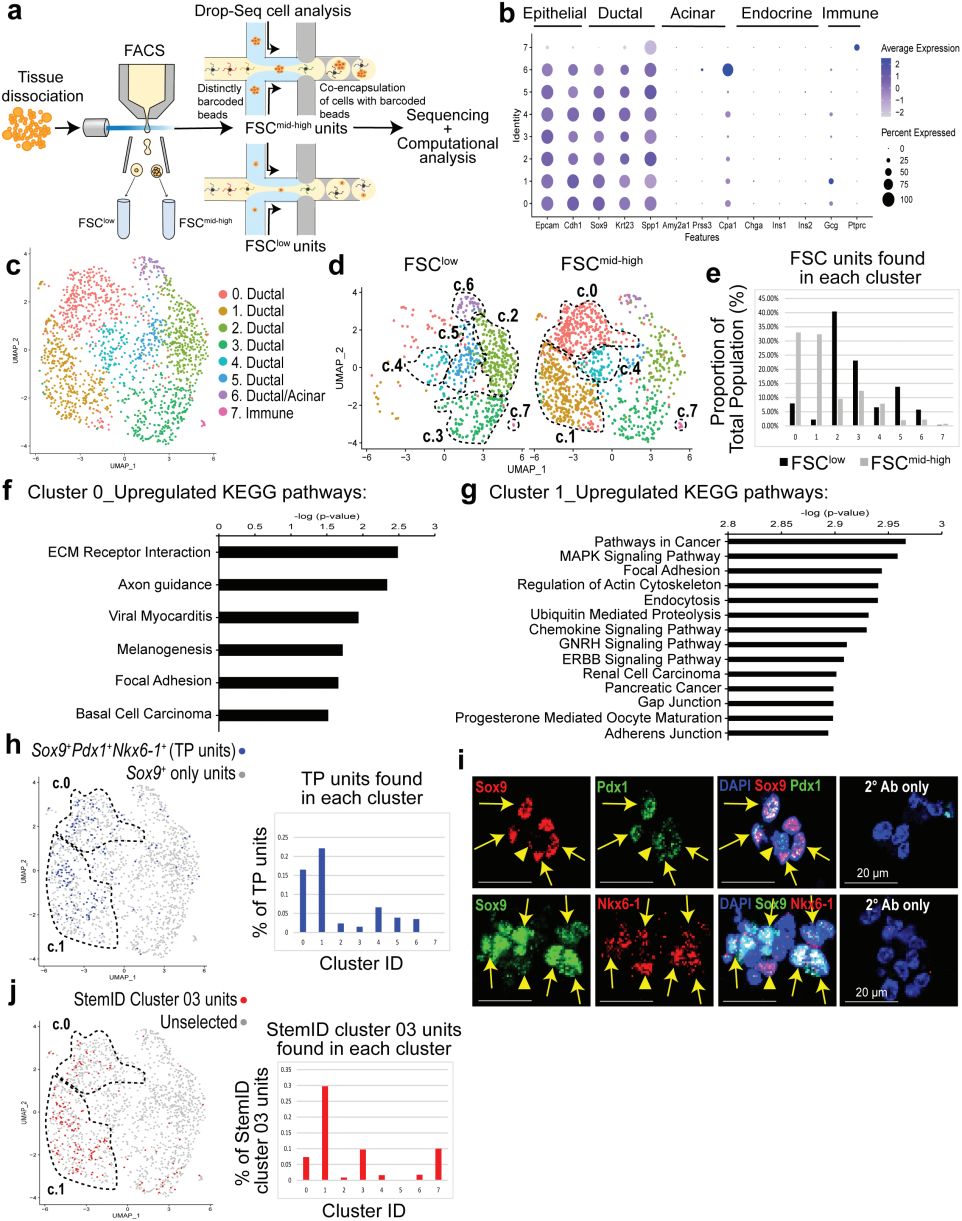
The ductal CD133^high^CD71^low^FSC^mid-high^ fraction expresses genes with a progenitor cell signature, co-expressing *Sox9, Pdx1*, and *Nkx6-1*. (**a**) Schematic for droplet RNA-seq (Drop-Seq) analysis. Datasets from CD133^high^CD71^low^FSC^low^ (FSC^low^; *n* = 598) and CD133^high^CD71^low^FSC^mid-high^ (FSC^mid-high^; *n* = 1125) fractions were combined and clustered using Seurat. (**b**) Average scaled expression (measured by average Pearson residual) of canonical markers for epithelial, pancreatic, and immune cell types. (**c**) Visualization of clusters using uniform manifold approximation and projection (UMAP) on the combined datasets. (**d**) The 2 datasets were split in UMAP. (**e**) Proportion of the FSC^low^ or FSC^mid-high^ units found in each cluster from total population. (**f–g**) Top pathways in cluster 0 or cluster 1, ranked by *P*-values, identified by gene set enrichment analysis (GSEA) using KEGG database. (**h**) Units that co-express *Sox9*, *Pdx1,* and *Nkx6-1* were identified as triple-positive (TP) units and labeled in the UMAP as darker dots (left). Percentage of TP units per cluster is presented (right). (**i**) IF staining of ductal small clusters. Full arrows indicate Sox9^+^ ductal cells that co-express Pdx1 or Nkx6-1. Arrowheads indicate Sox9^-^Pdx1^+^ cells (upper panel) or Sox9^-^Nkx6-1^+^ cells (lower panel). Scale bar = 20 µm. (**j**) Aggregated dataset was first processed using StemID algorithm and the resulting StemID cluster 03 was then back-fitted into the Seurat UMAP clusters 0 to 7 (left). Percentage of StemID cluster 03 units was highest in UMAP cluster 1 (right). Abbreviations: KEGG, Kyoto Encyclopedia of Genes and Genomes.

To gain further molecular insight into the small clusters, droplet-based RNA sequencing (droplet RNA-seq) with barcoding^45^ on sorted FSC^mid-high^ and FSC^low^ fractions was performed. After quality control (Supplementary Fig. S4a**–**S4c), a total of 1125 FSC^mid-high^ and 598 sorted FSC^low^ units were analyzed (Fig. 5a). These 2 sets of data were merged, and a total of 8 clusters were identified (Fig. 5b–5c, Supplementary Fig. 4d–4f). Consistent with our prior finding that the parent CD133^high^CD71^low^ population is comprised of ductal cells,^31^ most of the clusters shown by the uniform manifold approximation and projection (UMAP) (Fig. 5c) expressed ductal markers (*Sox9, Krt23, Krt17, Spp1*), except for cluster 7 which expressed immune cell genes (Fig. 5b, Supplementary Fig. S4g**–S4**i).

The FSC^mid-high^ and FSC^low^ fractions were enriched in UMAP clusters 0/1 and clusters 2/3/5/6, respectively (Fig. 5d–5e). Cluster-specific differentially expressed genes (Supplementary Dataset 1) were further analyzed by gene set enrichment analysis (GSEA) using the KEGG and gene ontology (GO) databases (Supplementary Datasets 2 and 3). The common top-upregulated GSEA-KEGG pathway among clusters 0 and 1 was focal adhesion (Fig. 5f–5g), suggesting interaction with ECM proteins.^46^ GSEA-KEGG also identified up-regulation in cancer and various signaling pathways (e.g., chemokine, GNRH, and ERBB) in cluster 1, but not in cluster 0 (Fig. 5f–5g). GSEA-GO analysis identified common pathways in both clusters 0 and 1, including cell junction organization, cell part morphogenesis, and response to wounding (Supplementary Fig. S5a, Dataset 2). In contrast, most up-regulated pathways among clusters 2, 3, 5, and 6 were involved in metabolism in GSEA-KEGG and GSEA-GO analysis (Supplementary Fig. S5a**–S5**b).

### The Ductal Small Clusters is Enriched for Genes With Progenitor Cell Signature

The presence of TP cells in 3-week-old Cystic colonies (Fig. 2k) prompted us to examine whether UMAP clusters 0 and 1 were enriched with TP units. Indeed, clusters 0 (16.5%) and 1 (22.1%) contained the highest proportions of TP units compared to clusters 2-7 (7% or less; Fig. 5h); IF staining of small clusters confirmed the Sox9^+^Pdx1^+^ and Sox9^+^Nkx6-1^+^ cells (Fig. 5i). Further analysis of other established embryonic MPC markers^47^ revealed that clusters 0 and 1 express *Hnf1b, Hes1, Foxa2, Tead1, Nkx2-2, Rbpj, Glis3, Nr5a2, Gata6, Yap1, Taz*, and *Myc*, but not *Ptf1a* and *Gata4* (Supplementary Fig. S6). The differentially expressed genes in TP units are presented in Supplementary Dataset 1. Taken together, these results demonstrate that clusters 0 and 1 are enriched for TP units.

To further explore the idea of progenitor cells, the StemID algorithm,^18^ a mathematical tool capable of identifying rare stem and progenitor-like cells within a heterogenous population of cells, was employed. StemID independently clustered the original dataset into 18 new clusters (Supplementary Fig. S7a); branch point analysis rendered a lineage tree that showed differentiation trajectories (Supplementary Fig. S7b). The StemID score for each cluster was then calculated (Supplementary Fig. S7c–S7h)^18^; StemID cluster 03 had the highest StemID score. The StemID cluster 03 was composed of 167 units (differentially expressed genes are presented in Supplementary Dataset 1), and was enriched in the FSC^mid-high^ compared to the FSC^low^ fraction (Supplementary Fig. S7i). Cluster 1 of the original dataset was most enriched for StemID cluster 03 (29.7%), compared to all other clusters (10% or less; Fig. 5j), indicating that cluster 1 is most enriched for progenitor-like cells.

To confirm the results from droplet RNA-seq, freshly sorted FSC^low^ and FSC^mid-high^ fractions, as well as presort cells, were analyzed by conventional qRT-PCR. Consistently, both fractions expressed higher levels of *Sox9* and lower levels of *Amylase2a* and *Insulin2*, compared to unsorted total pancreatic cells (Supplementary Fig. S8a–S8c).

### The Ductal Small Clusters Contain Tightly Bound Individual Cells Resistant to Enzymic Dissociation In Vitro

**Figure 6.**
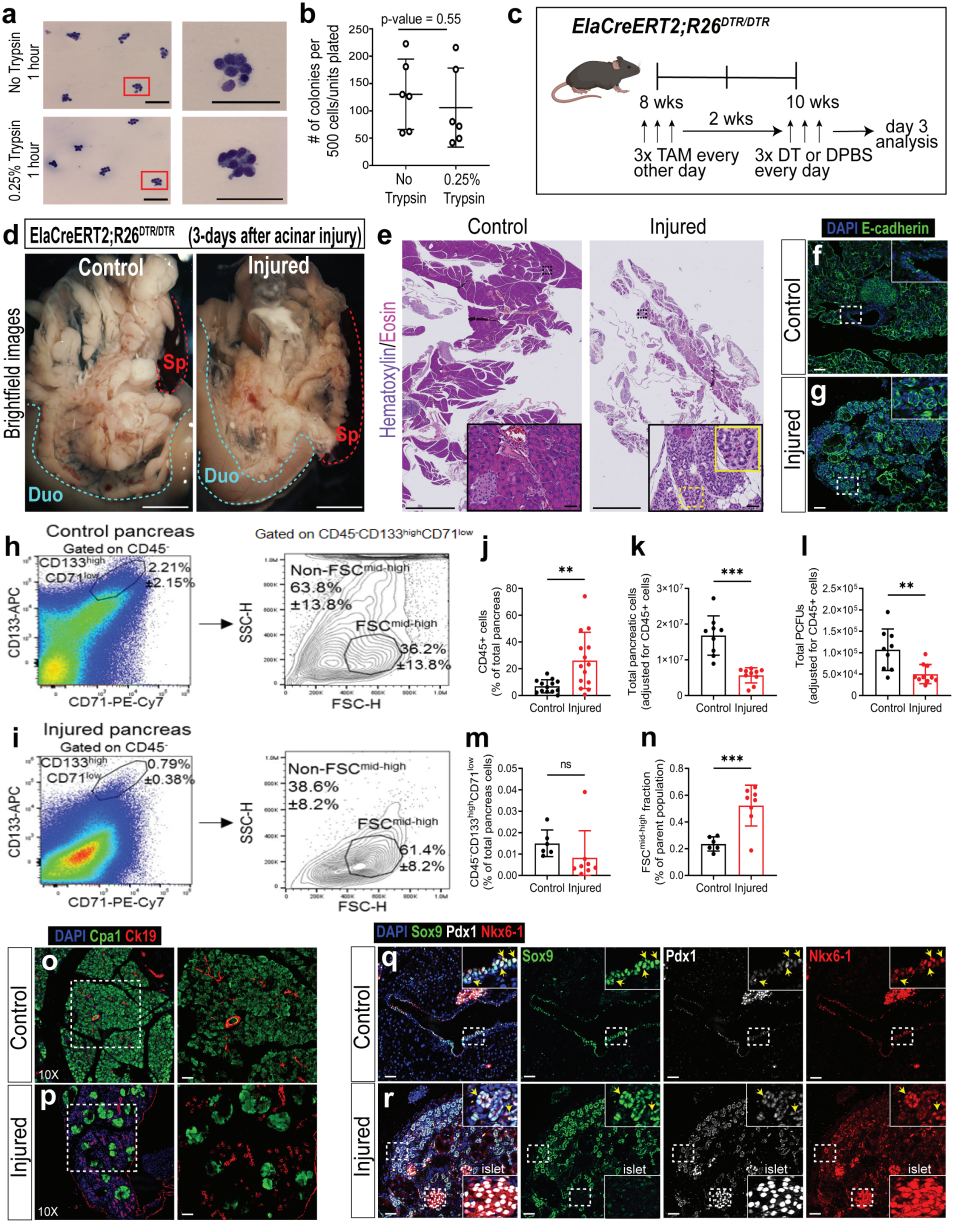
Ductal clusters resist trypsin digestion in vitro and preferentially survive in vivo after a severe acinar cell injury. (**a**) Sorted CD133^high^CD71^low^FSC^mid-high^ (FSC^mid-high^) fraction was incubated with DPBS vehicle (without trypsin; top) or with 0.25% trypsin (bottom) for 1 hour, followed by cytospin and Wright-Giemsa staining. Representative photomicrographs are shown. Scale bars = 100 µm. (**b**) Small clusters treated with or without trypsin for 1 hour were plated into the Matrigel/RSPO1 colony assay for 3 weeks and colony-forming efficiency determined. *n* = 3 independent experiments with 2 biological replicates each. Statistics were performed using 2-tailed Student’s *t*-test. Error bars represent SEM. (**c**) Schematic for in vivo acinar cell injury. *ElaCreERT2;R26^DTR/DTR^* mice were injected with tamoxifen (TAM) to induce the expression of diphtheria toxin receptor (DTR) in acinar cells, followed by injection of diphtheria toxin (DT) to ablate acinar cells. (**d**) Representative brightfield images of control and injured pancreas 3 days after the last DT injection. Abbreviations: Duo, duodenum; Sp, spleen. Dotted lines outline duodenum or spleen. Scale bars = 1 mm. (**e**) Representative images of H&E staining. Dashed lines outline ductal clusters. Scale bars = 2.5 mm. (**f–g**) IF staining of epithelial marker E-cadherin. Scale bars = 50 µm. (**h–i**) Representative flow cytometry of the parent CD133^high^CD71^low^ ductal population after gating on CD45-negative cells, followed by FSC analysis. (**j**) % CD45^+^ cells increased in the injured pancreata compared to controls. *n* = 13-14 mice/group. (**k**) The total number of cells per pancreas, after adjusting for % CD45^+^ cells, was reduced in the injured pancreata compared to controls. *n* = 9-10 mice/group. (**l**) Unsorted cells from control and injured pancreata were plated into Matrigel/RSPO1 colony assay to determine colony-forming efficiency, and subsequently the total number of PCFUs per pancreas was calculated. *n* = 9-10 mice/group. (**m**) % CD133^high^CD71^low^ ductal population after gating on CD45-negative cells. *n* = 6-8 mice/group. (*n*) % CD133^high^CD71^low^FSC^mid-high^ fraction among the CD133^high^CD71^low^ parent population. *n* = 6-8 mice/group. Statistics were performed using 2-tailed Student’s *t*-test. Error bars represent SD. (**o–p**) IF staining of formalin-fixed paraffin-embedded pancreas tissue slides with acinar (Cpa1) and ductal (Krt19) markers. Images on right are 10x magnified. Scale bars = 50 µm. (**q–r**) IF staining of Sox9, Pdx1, and Nkx6-1; arrows indicate cells co-expressing these 3 proteins. Islets served as the positive control for Pdx1 and Nkx6-1 staining. Scale bars = 50 μm.

Since the small clusters were grouped with multiple cells (Fig. 1b), we sought to dissociate them into single cells. Because we knew that small clusters were resistant to collagenase (which was used to dissociate the pancreas prior to sorting), trypsin was used. Surprisingly, small clusters remained intact even after a 1 hour incubation with 0.25% trypsin-EDTA (Fig. 6a) and retained colony-forming ability compared to untreated small clusters (Fig. 6b). Other enzymes, including Liberase and TrypLE, were tested with similar results (data not shown). The resistance to trypsin dissociation was likely due to cell-adhesion properties^48^; therefore, tight junction markers E-cadherin, TJP1 (ZO-1), and F11r (JAM-A)^49,50^ were examined, which were found at cell-cell interfaces of the small clusters by IF analysis (Supplementary Fig. S9a). In contrast, 3-week-old Cystic colonies expressed low levels of ZO-1 and JAM-A (Supplementary Fig. S9b–S9c). Transmission electron microscopy also revealed tight junctions at cell-cell boundaries of individual cells within the small clusters (Supplementary Fig. S9d, arrows), and each cell in a small cluster was single-nucleated (Supplementary Fig. S9d, nuclei labels). Counting the number of nuclei per cluster in Giemsa-Wright staining revealed that small clusters had an average of 8 cells (Supplementary Fig. S9e-S9f). To visualize individual cells within the small clusters in 3D space, serial block-face 3D scanning electron microscopy (3D-SEM) was employed, which confirmed that small clusters are comprised of multiple individual cells (Supplementary Movie S3). Taken together, these results demonstrate that small clusters consist of individual single-nucleated cells that are tightly bound and resist trypsin digestion.

### The CD133^high^CD71^low^FSC^mid-high^ Fraction Preferentially Survives 3 Days After Ablation of Acinar Cells In Vivo

The finding that small clusters remained intact after prolonged trypsin treatment in vitro raised the possibility that they may survive in adult mice after damage to acinar cells, which can release digestive enzymes in situ.^51^ To test this, the *ElaCreERT2;R26^DTR/DTR^* mice were employed to conditionally ablate acinar cells in vivo.^34^ The *ElaCreERT2;R26^DTR/DTR^* mice were injected with tamoxifen (TAM) to induce the expression of diphtheria toxin receptor (DTR) in acinar cells, followed by treatment with high doses of diphtheria toxin (DT) to ablate acinar cells (Fig. 6c). Three days after the last dose of DT, the body weight was unchanged (Supplementary Fig. S10a), but pancreata from injured mice were smaller in size compared to controls (Supplementary Fig. S10b). Additional control mice did not show a reduction in pancreas weight (Supplementary Fig. S10c). Translucent patches, indicative of edema, in both the head and tail of the pancreas were found in the injured pancreas (Fig. 6d). H&E staining further confirmed acinar cell loss (Fig. 6e), and the remaining cells in the injured pancreas were still E-cadherin-positive epithelial cells (Fig. 6f–6g).

Immune cells are known to migrate to the pancreas after severe damage^52^; thus, the proportion of infiltrating CD45^+^ leukocytes was determined using flow cytometry (Fig. 6h-ISupplementary Fig. S11a–S11f), which was found to increase in the injured pancreas compared to control (Fig. 6j). Therefore, CD45^+^ cells were gated out for subsequent flow cytometry analyses. The total number of pancreatic cells in each mouse was lower in the acinar-ablated pancreas compared to controls (Fig. 6k), confirming injury. Next, colony-forming efficiency was determined from each pancreas by plating into the Matrigel/RSPO1 colony assay for 3 weeks. Using the total number of pancreatic cells and colony-forming efficiencies in each pancreas, the calculated total number of PCFUs per pancreas was reduced in the injured pancreata compared to controls (Fig. 6l), suggesting that at least some PCFUs were lost after acinar cell ablation. Although trending lower, the proportion of the parent CD133^high^CD71^low^ population among the total pancreatic cells in the injured pancreata was not different compared to controls (Fig. 6m), suggesting that this ductal subpopulation was maintained. However, the proportion of the FSC^mid-high^ compared to the other FSC fractions within the parent CD133^high^CD71^low^ cell population was increased in the injured pancreata compared to controls (Fig. 6n). The results were similar between male and female cohorts (Supplementary Fig. S12). Together, these data demonstrate that ductal clusters preferentially survive after acinar cell injury in vivo.

Interestingly, epithelial cells clustered as rosettes were observed in injured pancreas (Fig. 6e–6g). We previously used the same *ElaCreERT2;R26^DTR/DTR^* mice to study acinar cell regeneration but with a lower DT dose.^34^ The clusters were also present in the *ElaCreERT2;R26^DTR/DTR^* mice treated with low doses of DT (Supplementary Fig. S10d, i-iii insets).

Acinar cell injury often leads to acinar-to-ductal cell metaplasia (ADM), which can be detected by co-expression of acinar and ductal markers.^53^ IF staining using Ck19 and Cpa1 (Fig. 6o-6p) as well as Ck19 and amylase (Supplementary Fig. S10e–10f) at 3 days post-acinar cell injury revealed that clusters expressed Ck19 but not Cpa1 or amylase, and thus are unlikely the products of ADM.

### Ductal Clusters That Survive Acinar Cell Ablation Express Sox9, Pdx1, and Nkx6-1, and Proliferate 14 Days Post-Injury

The TP cells found in small clusters (Fig. 5i) prompted us to examine TP cells in the endogenous and injured pancreas. In control mice, TP cells were found in the main, small interlobular, and intercalated ducts (Fig. 6q, Supplementary Fig. S10g). In injured mice, TP cells were found predominantly in the clusters 3 days post-injury (Fig. 6r).

**Figure 7.**
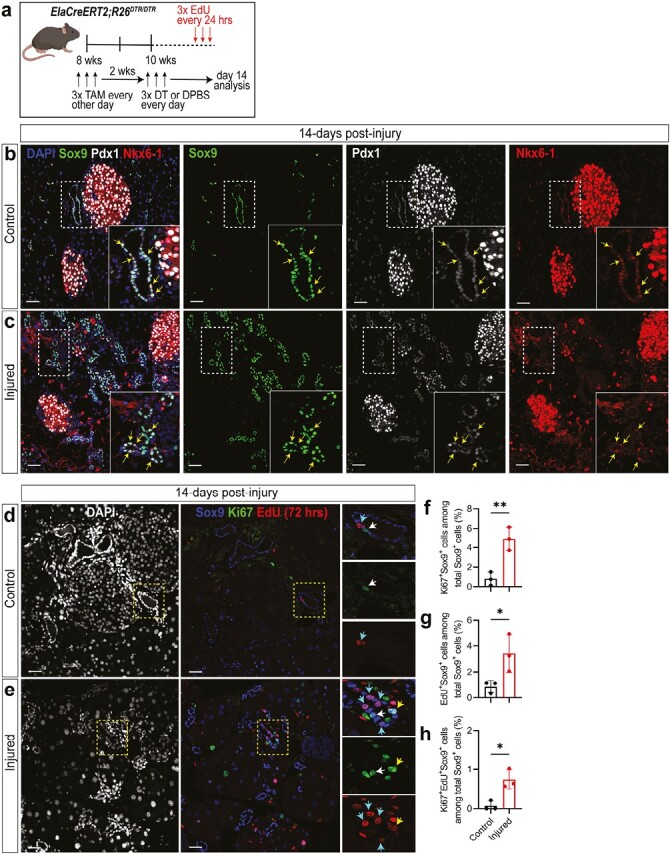
Ductal clusters continue to co-express Sox9, Pdx1, and Nkx6-1 and start to proliferate 14 days after severe acinar cell injury. (**a**) Experimental scheme for in vivo acinar cell injury and EdU treatment. (**b–c**) IF staining of Sox9, Pdx1, and Nkx6-1 in pancreas from control mice or 14 days after acinar cell ablation. (**d–e**) IF staining of Sox9, Ki67, and EdU. Scale bars = 50 μm. (**f–h**) Quantification of Sox9^+^ ductal cells that co-express Ki67 (f), EdU (g) or both (h) in control and injured pancreas. *n* = 3 mice/group. Statistics were performed using 2-tailed Student’s *t*-test. Error bars represent SD.

To determine the proliferation status of the ductal clusters, a thymidine analog 5-ethynyl-2ʹ-deoxyuridine (EdU) was injected into mice for 3 consecutive days before procurement of the pancreas (Supplementary Fig. S13a). Although some cells labeled with EdU on day 3 post-injury were detected, they were not in the clusters (Supplementary Fig. S13b–S13c). Therefore, the pancreas was examined 14 days post-injury (Fig. 7a); pancreas weight remained reduced (Supplementary Fig. S14a–S14c) and the ductal clusters were still present (Supplementary Fig. S14d–S14e). Importantly, the ductal clusters continued to express Sox9, Pdx1, and Nkx6-1 (Fig. 7b–7c) and were now positive for EdU or Ki67 (a marker for active proliferating cells) (Fig. 7d–7e, Supplementary Dataset 4). IF staining and quantification of total Sox9^+^ ductal cells confirmed increased Ki67^+^ (Fig. 7f), EdU^+^ (Fig. 7g), and Ki67^+^EdU^+^ cells (Fig. 7h) in injured compared to control pancreata. Again, no sign of ADM was detected among the ductal clusters (Supplementary Fig. S14f-S14k). Taken together, these findings demonstrate that ablation of acinar cells in vivo results in ductal clusters organized as rosettes that express Sox9, Pdx1, and Nkx6-1, and the ductal clusters become proliferative 14 days post-injury.

## Discussion

In this study, we discover that the tightly bound, multi-cellular, ductal cell clusters are the fundamental units of progenitor-like cells in adult mice. They are obtainable after the pancreas is dissociated by collagenase followed by sorting with the CD133^high^CD71^low^FSC^mid-high^ gate. They represent only ~0.1% of the normal pancreatic cells, possess self-renewal and tri-lineage differentiation potentials, preferentially survive acinar cell injury, and become proliferative 14 days post-injury. To the best of our knowledge, such ductal cell clusters have not been described before in the literature.

Our finding adds to the growing number of studies showing that adult pancreatic ductal cells are highly heterogeneous not only in humans^17-20^ but also in mice.^21^ We previously showed that in 2-4 months old mice, CD133^+^ ductal cells comprise 13.1 ± 4.3% of the total pancreatic cells.^35^ The CD133^high^CD71^low^ ductal subpopulation comprises 2.4 ± 1.9% of the total pancreatic cells.^35^ In this study, the ductal CD133^high^CD71^low^FSC^mid-high^ fraction is only 0.12 ± 0.04% of the total pancreatic cells, but is highly enriched for progenitor-like cells. With such a small incidence of cells, researchers who use pan-ductal markers such as HNF1b^15^ or Sox9^16^ to conduct in vivo lineage-tracing experiments should expect minor events in the resulting datasets. In addition, droplet RNA-seq demonstrates the diversity of gene expression patterns among various ductal cell groups (Fig. 5). The clonal selection of a progenitor cell within a single small cluster gives rise to a colony and the unequal sizes of the resulting colonies (Fig. 4) further demonstrate the heterogeneity of the ductal cells even within the small clusters.

The finding that the ductal small clusters retain colony-forming ability even after 1 hour of trypsin digestion in vitro (Fig. 6a–6b) was unexpected. We found only one other example in the literature on normal pituitary gland from which small clusters of progenitor cells are also resistant to trypsin digestion in vitro while maintaining the ability to form colonies that express pituitary gland hormones.^54^ The biological significance of the clustering of normal cells is currently unknown. However, it has been shown that cancer cells cluster to reduce reactive oxygen species and promote cell survival.^55^ Given the potentially harsh microenvironment of the ductal progenitor cells where digestive enzymes are abundantly available, the clustering of these ductal cells may provide a survival advantage amid insults such as pancreatitis. Although at the steady state the pancreas is largely quiescent,^1^ pancreatitis is known to induce various pancreatic cells to assist in repair and regeneration.^56^ Consistent with this body of prior work, our results confirm that ductal cells become proliferative in response to acinar cell injury (Fig. 7d–7h).

One new and significant observation from this study is that murine adult ductal clusters are triple positive for Sox9, Pdx1, and Nkx6-1, and they preferentially remained after acinar cell injury. We recently reported the existence of SOX9^+^/PDX1^+^/NKX6-1^+^ progenitor cells in the endogenous ducts of normal adult human pancreas.^20^ The TP cells are a feature of embryonic MPCs^32,33^ but have been unknown in the adult pancreas. In addition, adult ductal clusters express many other known embryonic MPC genes, such as *Hnf1b, Hes1, Foxa2, Tead1, Nkx2-2, Rbpj, Glis3, Nr5a2, Gata6, Yap1, Taz*, and *Myc*^47^; interestingly, *Ptf1a* and *Gata4* are absent (Supplementary Fig. S6). In mice, while both Gata4 and Gata6 are required for pancreas organ development,^57^ Gata4 is known to be more competent than Gata6 in rescuing early developmental defects in the absence of the other Gata factor.^58^ Ptf1a is co-expressed with Pdx1 in both murine^59^ and human^60^ embryonic MPCs and is also required for pancreas organogenesis.^59,61^ The biological significance for the lack of Gata4 and Ptf1a in adult ductal clusters is currently unknown, but may highlight differences between embryonic versus adult MPCs that may lead to a differential regenerative response. Intriguingly, pathways involved in responses to wounding, DNA damage, and proinflammatory cytokines, such as interleukin 6, interferon-gamma (Supplementary Dataset 2; c1), and chemokine (Fig. 5g) are upregulated in UMAP cluster 1. These results may suggest that adult ductal progenitor cells are equipped to react to inflammation—an idea supported by a recent finding that an elevated level of HLA-DR, a key molecule in adaptive immunity, is observed in human ductal cells from donors with type 1 diabetes.^62^

Of note, we find that genes in the pancreatic cancer pathway are upregulated in the ductal clusters (Fig. 5g). This reinforces the idea that progenitor features are correlated with tumorigenesis.^63^ Other groups have shown that ductal cells can initiate pancreatic ductal adenocarcinoma.^64,65^ Our current results raise the possibility that the survival advantage of these ductal clusters post-injury may render them amenable to transformation over the lifespan of the organism. Thus, the adult ductal clusters identified here have implications in tumorigenesis as well.

In summary, we have identified rare multi-cellular clusters in the adult mouse pancreatic ducts capable of (1) self-renewal and tri-lineage differentiation in vitro, and (2) surviving and proliferating after acinar cell injury in vivo. These cell clusters express not only conventional ductal markers but also many embryonic MPC markers. We recently showed that human adult ductal progenitor-like cells can be differentiated into endocrine progenitor cells in vitro using a Notch signaling inhibitor, and these endocrine progenitor cells subsequently give rise to insulin expressing cells that function in transplanted insulin-dependent diabetic mice.^20^ Together, these results implicate adult ductal progenitor-like cells as potential targets for beta cell neogenesis and regenerative medicine in diabetes.

## Supplementary Material

Supplementary material is available at *Stem Cells* online.

sxae005_suppl_Supplementary_Data

## Data Availability

Raw data for droplet RNA sequencing are available at the Gene Expression Omnibus (GEO) database (code GSE249084). All other data underlying this article will be shared on reasonable request to the corresponding author.
