# Supplementary material for: Rare, Tightly-Bound, Multi-Cellular Clusters in the Pancreatic Ducts of Adult Mice Function Like Progenitor Cells and Survive and Proliferate After Acinar Cell Injury
Source: Stem Cells. 2024 Jan 11;42(4):385–401. doi: 10.1093/stmcls/sxae005 (PMC11016848; doi:10.1093/stmcls/sxae005)
Supplement: sxae005_suppl_Supplementary_Data [file sxae005_suppl_supplementary_data.zip › sxae005/Supplementary datasets and movie description_5_9_2022_11_21_2023_12_19_2023.docx]

**Description of Additional Supplementary Files**

Title: **Supplementary Dataset 1**

Description: Cluster-specific, differentially expressed genes among the 8 clusters presented in Figure 6c are identified and presented. In addition, differentially-expressed genes between Sox9+/Pdx1+/Nkx6-1+ (TP) cells versus the non-TP cells (Figure 5h), as well as Stem ID cluster 3 versus non STEM ID cluster 3 (Figure 5j) are presented.

Title: **Supplementary Dataset 2**

Description: Differentially expressed genes among the 8 clusters presented in Figure 5c were further analysed by gene set enrichment analysis (GSEA) using Gene Ontology (GO) molecular signature databases.

Title: **Supplementary Dataset 3**

Description: Differentially expressed genes among the 8 clusters presented in Figure 5c were further analyzed by gene set enrichment analysis (GSEA) using KEGG molecular signature databases.

Title: **Supplementary Dataset 4**

Description: Data associated with Figure 7 f-h. Quantification of proliferating Sox9+ ductal cells in the pancreas of control versus acinar cell injured mice 14 days after the last high dose of diphtheria toxin injection.

Title: **Supplementary Movie 1**

Description: CD133^high^CD71^low^FSC^mid-high^ small clusters were plated into Matrigel/RSPO1 colony assay for 3 weeks. The resulting Cystic colonies were subjected to whole-mount immunostaining with antibodies against Amylase or EpCAM, followed by confocal imaging analysis. Movie is stitched from sequential Z-stack images. Green, EpCAM; Red, Amylase.

Title: **Supplementary Movie 2**

Description: CD133^high^CD71^low^FSC^mid-high^ small clusters were plated into laminin colony assay for 10 days. The resulting “Endocrine/Acinar (E/A)” colonies were subjected to whole-mount immunostaining with antibodies against C-peptide or glucagon, followed by confocal imaging analysis. Movie is stitched from sequential Z-stack images. Red, C-peptide; Green, Glucagon.

Title: **Supplementary Movie 3**

Description: Individual cells are found in a CD133^high^CD71^low^FSC^mid-high^ small cluster. Serial block-face scanning electron microscopy (SBF-SEM), a.k.a. 3D-SEM, was used to generate 500 serial images of a cluster. Using Amira software, the images were stitched sequentially and presented as a movie. The movie starts by scanning through the cells from the top of the stacked images to the bottom, then scans from the bottom of the stacked images to the top. The borders of individual cells were segmented; each cell was painted a unique colour. Translucent colours were rendered initially and then changed to solid colours to increase visualization of the arrangement of cells. No multi-nucleated cells were found.
