# Supplementary material for: Rare, Tightly-Bound, Multi-Cellular Clusters in the Pancreatic Ducts of Adult Mice Function Like Progenitor Cells and Survive and Proliferate After Acinar Cell Injury
Source: Stem Cells. 2024 Jan 11;42(4):385–401. doi: 10.1093/stmcls/sxae005 (PMC11016848; doi:10.1093/stmcls/sxae005)
Supplement: sxae005_suppl_Supplementary_Data [file sxae005_suppl_supplementary_data.zip › sxae005/Supplementary figures and tables_5_9_2023-3_11_22_2023_12_19_2023.pdf]

Supplementary Information

Title: Rare, tightly-bound, multi-cellular clusters in the pancreatic ducts of adult mice function like progenitor cells and survive and proliferate after acinar cell injury

Authors: Jacob R. Tremblay*, Jose A. Ortiz*, Janine C. Quijano, Heather Zook, Neslihan Erdem, Jeanne M. LeBon, Wendong Li, Kevin Jou, Walter Tsark, Jeffrey R. Mann, Mark Kozlowski, David A. Tirrell, Farzad Esni, Dannielle D. Engle, Arthur D. Riggs, Hsun Teresa Ku

*equal contribution

This document contains:

Supplementary Materials and Methods

Supplementary Figures 1-14

Supplementary Tables 1-2

Reference Cited

SUPPLEMENTARY MATERIALS & METHODS

Mice. Animal experiments were conducted according to the Institutional Animal Care and Use Committee at the City of Hope. C57BL/6J (B6) mice (The Jackson Laboratory, Bar Harbor, ME) (both sexes) were used in most experiments unless specified otherwise. Transgenic Ela-CreERT2;R26$^{DTR/DTR}$ mice were as reported previously [1] but were crossed to B6 mice to yield >99.99% B6 genetic background. FVB.129S1-Hprt$^{tm1(CAG-DsRed)Mnn/COH}$ (*Hprt*$^{DsRed/+}$) mice were generated in-house using gene targeting vectors and embryonic stem (ES) cell clone selection strategies. The coding sequence (cds) of the T1 variant of *Discosoma* sp. red fluorescent protein (DsRed), also known as DsRedExpress [2], was obtained from Clontech and knocked-in at the X-linked hypoxanthine guanine phosphoribosyl transferase (*Hprt*) locus, as previously described [3], except: (1) the DsRed cds was used in place of the Cre cds, and (2) *lox*P sites flanked the neo selection cassette. Neomycin (neo)-resistant clones were genotyped using Southern blots to identify putative *Hprt*$^{DsRed/+}$ 129S1 recombinants. *Hprt*$^{DsRed/+}$ male chimeras were mated to 129S1/SvImJ (129S1) females to confirm germline transmission of the transgene. The neo selection cassette was removed by breeding with 129S1 Cre deleter mice [3] to produce the final knock-in line. Subsequently, Hprt$^{DsRed/+}$ mice were bred and phenotyped using visual observation of red fluorescence with a TRITC/Cy3 filter set for excitation 454 ±15 nm and emission 620 ± 30 nm wavelength. *Hprt*$^{DsRed/+}$ 129S1 mice were back-crossed to FVB/NJ mice for >10 generations to produce an *Hprt*$^{DsRed/+}$ 129S1.FVB/NJ congenic strain to improve fluorescence imaging in live mice and increase average litter size. All experiments were conducted using mice between 8-12 weeks of age.

Dissociation of pancreas. For each experiment using normal mice, a total of 5 pancreata were dissected, cleared of fat tissue under a dissecting microscope, and rinsed three times in cold Dulbecco's phosphate-buffered saline (DPBS) containing 0.1% bovine serum albumin (BSA), 100 Units (U)/mL penicillin, and 100 µg/mL streptomycin (referred to as DPBS/BSA). Pancreata were minced in a dry petri dish on ice using spring scissors for 3 min or until finely minced, transferred to a 50 mL conical tube, resuspended in DPBS/BSA containing collagenase B (2-4 mg/mL) (Roche, Mannheim, Germany) and DNase I (2,000 U/mL) (Calbiochem, Darmstadt, Germany) and incubated at 37°C for 16 min, with mixing every 2-3 min and

gently passing through a 16G syringe every 8 min. Dissociated pancreatic cells were washed in cold DPBS/BSA containing 2,000 U/mL DNase I and successively passed through 100 μm and 40 μm mesh filters (BD Biosciences, San Jose, CA) to yield a single-cell suspension.

Sorting and flow cytometry analysis. Sorting was performed as previously described [4]. Dissociated pancreatic cells/units were incubated with anti-mouse CD16/32 (10 μg/mL; BioLegend, San Diego, CA) for 5 min on ice to reduce non-specific binding, treated with biotin-conjugated anti-mouse CD133 (clone 13A4; 5 μg/mL; eBioscience, San Diego, CA) and phycoerythrin/Cy7 (PECy7)-conjugated anti-mouse CD71 antibodies (clone RI7217; 5 μg/mL; BioLegend, San Diego, CA) for 20 min on ice, washed twice, incubated with streptavidin-labeled allophycocyanin (APC) (2 μg/mL BioLegend) for 15 min on ice, washed twice, and resuspended in DPBS/BSA/DNase I containing DAPI (0.2 μg/mL). Control antibodies were biotin-conjugated rat immunoglobulin (Ig) G1 (5 μg/mL; eBioscience, San Diego, CA) and PE/Cy7-conjugated rat IgG1 (5 μg/mL; BioLegend, San Diego, CA). Flow cytometry data were collected using Fortessa LSRII (Becton Dickinson, San Jose, CA) or Attune NX Cytometer (Thermo Fisher, Waltham, MA). Sorting was performed on an Aria special order research product (SORP) (Becton Dickinson, San Jose, CA). All data were analyzed using FlowJo software (TreeStar, Ashland, OR).

Colony Assay. Sorted cells/units were resuspended at a density of 500 cells/units per well per 0.5 mL for the Matrigel/RSPO1 colony assay or 8.0×10^3 cells/units per well per 0.5 mL for the laminin hydrogel assay as described previously [5]. Culture media contained DMEM/F12 media, 1% (w/vol) methylcellulose (Shin-Etsu Chemical, Tokyo, Japan), 50% conditioned media from mouse embryonic stem cell-derived pancreatic-like cells [6] , 5% fetal calf serum (FCS), 10 mmol/L nicotinamide (Sigma, St. Louis, MO), 10 ng/mL human recombinant activin B (R&D Systems, Minneapolis, MN), 0.1 nmol/L exendin-4 (Sigma), and 1 ng/mL vascular endothelial growth factor-A (VEGF) (Sigma). When indicated, either 5% (vol/vol) Matrigel plus 750 ng/mL mouse recombinant RSPO1 (R&D) or 100 μg/mL of laminin hydrogel [5] was added. Cells/units were plated in 24-well ultra-low protein-binding plates (Corning, New York, USA) and incubated in a humidified 5% CO_2 atmosphere at 37°C. Colonies grown in Matrigel/RSPO1 or laminin assay were counted 2-3 weeks or 10 days after plating, respectively.

Micro-manipulation of single cells/units or colonies. Sorted cells/units were visualized under a phase-contrast microscope, individually lifted using a fine Pasteur pipet with a ~30 µm diameter opening, and placed into the Matrigel/RSPO1 colony assay at a concentration of 1 cell/unit per well in a low-attachment Nunclon 96-well plate (ThermoFisher) [7]. For micro-manipulation of single colonies, colonies grown in the 24-well plate were individually lifted using a 10-µL Eppendorf pipette set to 2 µL under direct microscopic visualization.

Serial dissociation and replating of colonies. Colonies grown in Matrigel/RSPO1 assay were collected in a 50 mL conical tube containing warmed DPBS/BSA (1 mL/well), washed, resuspended in 10 mL of 2-4 mg/mL collagenase B, incubated for 15 min at 37°C with mixing every 5 min, and washed in DPBS/BSA. Subsequently, colonies were treated with 20 mL of 0.25% (wt/vol) trypsin-EDTA, incubated for 3 min at 37°C, pipetted thoroughly and treated with warmed FCS (4 mL) to stop trypsin digestion. Cells were washed in DPBS/BSA and kept at room temperature. A sample was mixed with 0.02% (wt/vol) trypan blue, and the concentration of live cells (trypan blue-negative) was determined using a hemocytometer. Another portion was replated into Matrigel/RSPO1 colony assay as indicated above. The final total number of expanded PCFUs was calculated by multiplying the previous dilution factor(s) with the number of colonies per well in the present culture.

Cytospin and Wright-Giemsa Staining. A total of 2,500 cells/units from each sorted fraction was mixed with 150 µL FCS, added to the EZ single Cytofunnel fitted with Superfrost Plus microscope slide (ThermoFisher), and spun at 1,400 rpm for 5 min on a Shandon Cytospin 4 centrifuge system (ThermoFisher, Waltham, MA). For trypsin digestion experiment, sorted $FSC^{mid\text{-}high}$ fraction was treated with 0.25% trypsin-EDTA in low-binding 96-well plates at 2,500 units per well and incubated at 37°C for 1 hr. The reaction was stopped by adding 100% FCS, and the cells/clusters were spun onto slides. Slides were removed, air-dried for 5 min, and incubated with ice-cold 100% methanol for 5 min to fix cells/clusters. Subsequently, cells/clusters were stained with 10% modified Wright-Giemsa solution (Sigma) for 30 min, washed with water, and imaged using a Zeiss Observer II (ZEISS, Oberkochen, Germany).

Conventional or Microfluidic Quantitative Reverse Transcription-Polymerase Chain Reaction (qRT-PCR). Conventional and microfluidic qRT-PCR analyses were as reported [5]. For microfluidic qRT-PCR analyses, single colonies were micromanipulated, as described above, collected in reaction buffer (10 µL), and pre-amplified (12 or 18 cycles for Matrigel/RSPO1- or laminin-grown colonies, respectively) according to the manufacturer's instructions (Fluidigm). Pre-amplified cDNA was loaded onto a 48.48 Dynamic Array using a NanoFlex integrated fluidic circuit (IFC) controller (Fluidigm), and thermocycling performed using the BioMark™ system (Fluidigm, South San Francisco, CA). Threshold cycle (Ct) was determined using BioMark PCR analysis software (Fluidigm) and expressed as delta Ct against the internal control beta-actin. All experiments were performed with negative (water) and positive controls (adult B6 pancreatic cells). Taqman probes used are listed in **Supplementary Table 1**.

Droplet-based RNA-sequencing. $CD133^{high}CD71^{low}FSC^{mid\text{-}high}$ and $CD133^{high}CD71^{low}FSC^{low}$ fractions were sorted, counted, and diluted in DPBS supplemented with 0.1% BSA to a concentration recommended by the manufacturer. Cell/units were captured on a 10x Chromium device using a 10X V3 Single Cell 3' Solution kit (10x Genomics, Chromium Single Cell 3' Regent 00kit V3 Chemistry, Cat. PN-1000092). All protocols were performed following the manufacturer's instructions. Final sequencing libraries were analyzed on a High Sensitivity DNA Chip (Agilent, Cat 5067-4626) to determine library size; final library concentrations were determined using a Qubit High Sensitivity DNA Assay Kit (ThermoFisher). Libraries were sequenced using the paired end setting of 101-101 with 8 cycles of index reads on an Illumina NovaSeq 6000 platform. Approximately 0.1 million reads per cell/unit were sequenced.

Data analysis for droplet RNA-sequencing. Raw sequencing data were aligned to the mouse genome (mm10) and the R package Seurat was used for gene filtration, normalization, principal component analysis (PCA), variable gene finding, clustering analysis, and Uniform Manifold Approximation and Projection (UMAP) dimension reduction. A matrix containing gene-by-unit expression data was imported to create individual Seurat objects. Units with <200 detectable genes and >15% mitochondrial genes were excluded. Data were merged and log-normalized for subsequent analysis. PCA was performed for unbiased

clustering. Clusters were visualized with UMAP embedding. Differentially expressed genes between CD133^{high}CD71^{low}FSC$^{mid-high}$ and CD133^{high}CD71^{low}FSC^{low} samples in each cluster were determined using the function FindAllMarkers. Gene Ontology (GO) and Kyoto Encyclopedia of Genes and Genomes (KEGG) pathway analyses were performed on differentially expressed genes of each cluster using the gene set enrichment analysis (GSEA) function implemented in the clusterProfiler package; results were plotted using ggplot2.

Immunostaining of small clusters and colonies. Small clusters or colonies were fixed in 4% paraformaldehyde (PFA) containing 0.15% Triton X-100 at 4°C overnight, cryoprotected at 4°C overnight in 30% sucrose dissolved in PBS (pH = 7.4-7.6) containing 0.15% Triton X-100 (PBSX), followed by frozen embedding using Optimal Cutting Temperature (OCT) compound (ThermoFisher). Frozen blocks were sectioned (8 μm thickness) onto glass slides (Fisher Scientific), stored at -80°C, thawed at room temperature (RT), washed with PBSX and blocked with a PBS-based buffer containing 5% donkey serum and 0.1% Triton X-100 for 1 hr at RT. Slides were incubated with primary antibodies at 4°C overnight, washed, treated with secondary antibodies at RT for 2 hrs, washed and treated with VECTASHIELD Antifade Mounting Medium following the manufacturer's instructions (Vector laboratories, Burlingame, CA).

For whole-mount staining, Cystic colonies were placed in 1.5 mL Eppendorf tubes whereas E/A colonies in 96 U-bottom plate (ThermoFisher). Control samples were islets and islet-depleted exocrine tissues. Cells were fixed in 4% PFA containing 0.15% Triton X-100 for 1 hr, washed twice with PBSX, followed by blocking for 1 hr at RT with a filtered 10% donkey serum containing 0.1% Triton X-100 and Biogenex Laboratories Power Block (Fisher Scientific). Next, samples were incubated with primary antibodies at 4°C overnight, washed thrice using PBSX, and treated with secondary antibodies at 4°C overnight, followed by four washes with PBSX. Each wash is 10-15 min incubation at RT with gentle shaking, followed by centrifugation for 1 min at 300 g for Eppendorf tubes or 5 min at 400 g for a 96-well U-bottom plate. Importantly, gentle shaking was included for antibody treatments and washes. Finally, samples were incubated in 70-90% glycerol mounting solution (diluted in PBSX) at 4°C for 2 hrs or overnight before mounting onto glass slides for image analysis.

Images were captured on a Zeiss Axio-Observer-Z1 microscope (ZEISS), Zeiss LSM700, or Zeiss LSM880 with Aryscan and processed using Adobe Photoshop and Illustrator 2021. Antibodies used are listed in **Supplementary Table 2**.

Histology and staining of pancreas tissue. Pancreata were dissected, fixed in 10% formalin solution for 24 to 72 hrs, washed with PBSX, stored in 70% ethanol, paraffin embedded and sectioned (5 μm thickness) onto glass slides from the beginning to the end with representative slides taken 100 μm apart for subsequent quantification. Slides were prepared by baking at 56°C for 3 hrs, followed by de-waxing in xylene for 15 min, rehydrated in ethanol, and antigens retrieved using IHC-TekTM Epitope Retrieval Steamer Set (IHCWORLD) for 45 min in sodium citrate buffer (pH=5.5). Slides were then washed with PBSX, treated with permeabilization solution (0.3% Triton X-100 in PBS) for 30 min at room temperature (RT), washed, blocked for 2 hrs at RT with a buffer containing 5-10% donkey serum, 0.1% Triton X-100, and Biogenex Laboratories Power Block (Fisher Scientific), and incubated with primary antibodies at 4°C overnight followed by secondary antibodies at RT for 2 hrs. Slides were treated with Vector® TrueVIEW™ Autofluorescence Quenching Kit (Vector laboratories) following manufacturer's instructions to reduce autofluorescence and unspecific background signals. Nuclei were visualized by incubating slides for 15 min with 0.1 μg/ml 4,6-diamidino-2-phenylindole (DAPI, Thermo Fisher Scientific) in PBSX. For EdU detection, Click-iT EdU Alexa Fluor 555 Imaging Kit (Thermo Fisher Scientific) was used following the manufacturer's instructions. Finally, slides were washed with PBSX and treated with VECTASHIELD Antifade Mounting Medium following the manufacturer's instructions (Vector laboratories, Burlingame, CA). Images were captured with the ApoTome module on a Zeiss Axio-Observer-Z1 microscope (ZEISS) or a Zeiss LSM880 with Airyscan and processed using Adobe Photoshop and Illustrator 2021. Antibodies used are listed in **Supplementary Table 2**.

Transmission electron microscopy. $CD133^{high}CD71^{low}FSC^{mid\text{-}high}$ fraction was settled onto a glass cover slip pre-treated with poly-L-Lysine for 30 min at RT. Medium was gently removed and replaced with a fixative containing 0.15 M cacodylate buffer (pH 7.4), 2.5% glutaraldehyde and 2 mM calcium chloride. Small clusters on the cover slip were rinsed in a 0.15 M sodium cacodylate solution (pH 7.4) containing 2

mM calcium chloride, post-fixed in 1.5% potassium ferrocyanide-reduced 2% osmium tetroxide in 0.15 M cacodylate buffer for 1 hr, rinsed in distilled water and treated with 0.1% aqueous thiocarbohydrazide for 20 min. After further rinsing in distilled water, small clusters were treated with 2% osmium tetroxide for 30 min, rinsed in distilled water, dehydrated in ethanol, and infused with Durcupan ACM resin. Ultra-thin sections (~70 nm thick) were cut using a diamond knife on a Leica Ultracut UCT Ultramicrotome (Leica, Wetzlar, Germany) and placed on mesh copper EM grids. Images were captured using an FEI Tecnai 12 transmission electron microscope (ThermoFisher) equipped with a Gatan Ultrascan 2K CCD camera (Gatan, Pleasanton, CA).

Serial block-face 3D scanning electron microscopy (3D-SEM). Sample blocks were mounted on an aluminum pin and trimmed to 0.5 mm × 0.5 mm. Each specimen was placed in a Zeiss Sigma VP field-emission scanning electron microscope (ZEISS, Oberkochen, Germany) equipped with a serial block-face sectioning unit Gatan 3View (Gatan). A backscatter electron image of the face was obtained under an accelerating voltage of 4 keV and chamber pressure of 20 Pa under the Variable pressure mode. An automatic microtome removed a 70-nm thick slice from the sample and a new image was recorded. This procedure was repeated to yield a data set of 1,000 images from which a complete three-dimensional reconstruction was derived. Images were segmented, analyzed, and visualized using Amira software (ThermoFisher).

Quantification of DsRed^{+} cells in colonies from Hprt$^{DsRed/+}$ female mice. Images were taken using a Zeiss 700LSM confocal microscope (ZEISS). Samples were imaged using 1-µm optical sections. Optical slices from z-stack-imaged whole colonies were imported into Imaris (Bitplane, Zurich, Switzerland) and backgrounds were corrected. Nuclei diameters were set at 5 µm; red thresholds were set against negative and positive controls. Once parameters were set, a batch analysis was performed on all 32 colonies individually, and DAPI^{+}DsRed^{+} or DAPI^{+}DsRed^{-} cells were quantified using the above annotated batch results.

Proliferation analysis. Representative pancreatic sections (100 µm apart) were subjected to staining

described above and images were taken at 20X magnification with identical channel exposure time for control and injured samples. Images were processed using QuPath v0.2.3 software [8]. Briefly, object classification and single/composite measurement classifier from QuPath were used to quantify individual cells ($DAPI^{+}$) that co-expressed Sox9 plus Ki67 or Sox9 plus EdU. The parameters for object classification (i.e. pixel size, median radius, sigma, minimum area, maximum area, and threshold) were optimized using the Cell Detection algorithm. Subsequently, a script was generated to perform annotation measurements automatically for all images, and the annotation measurements were exported for analysis (**Supplementary Dataset 4**).

Acinar cell injury in mice. Tamoxifen (TAM) was injected intraperitoneally into ElaCreERT2;$R26^{DTR/DTR}$ or control mice at 0.2 mg/g body weight (b.w.) once per day, every other day, for a total of three injections. Three weeks later, a high dose of diphtheria toxin (DT) (200 ng/ 20g b.w.) was injected once per day for 3 days. For EdU labeling, control and injured adult mice were injected with EdU (100 mg/kg b.w., Abcam, Cambridge, UK) every 24 hrs for 3 days prior to the procurement of the pancreas. Mice were euthanized 3 or 14 days after the last DT injection. Control Cre-negative $R26^{DTR/DTR}$ mice received TAM and DT, and control ElaCreERT2;$R26^{DTR/DTR}$ mice received TAM and DPBS (vehicle for DT). Pancreata from individual mice were dissected and processed for paraffin embedding or cleared of fat tissue, dissociated with collagenase B as described above, and divided into three portions for 1) determining total cell number 2) flow cytometry with anti-CD45, anti-CD133 and anti-CD71 antibodies, and 3) plating into Matrigel/RSPO1 colony assay to determine colony-forming efficiency.

Statistical Analysis. GraphPad Prism 8 software was used for statistical analysis. For analysis between two groups with equal sample size, unpaired, two-tailed Student's *t*-test was used to determine statistical significance; those with unequal sample size, unpaired, two-tailed Student's *t*-test with Welch's correction was used. For analysis of more than two groups, one-way ANOVA followed by Tukey post hoc analysis was used. Data format, presented as mean ± SD or mean ± SEM, and sample size are indicated in the figure legend. Significance is defined as *p<0.05, **p<0.01, ***p<0.001, ****p<0.0001.

Data Availability. All underlying data is available to interested researchers within reason.

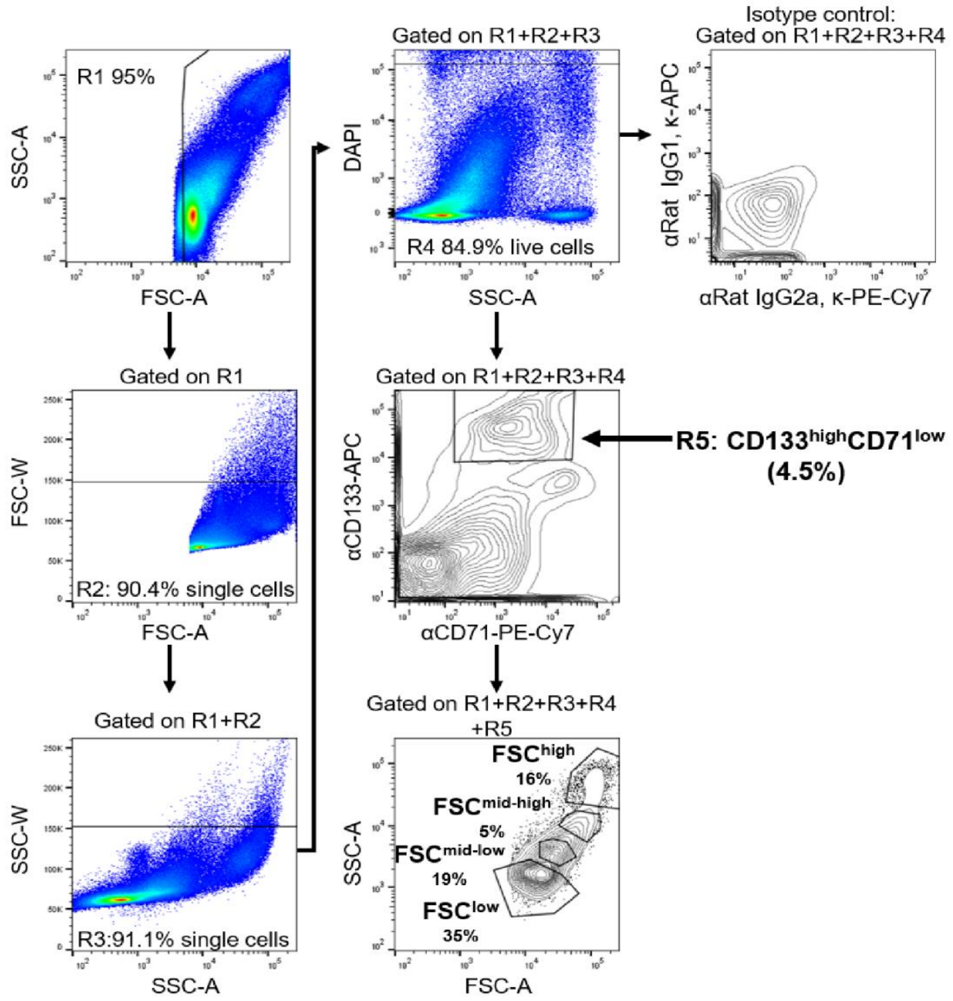

Supplementary Figure 1. Gating strategy for the ductal CD133^{high}CD71^{low} population, associated with Figure 1.

Pancreatic cells were stained with anti-CD133 and anti-CD71 antibodies and sequentially gated using the following regions (R). R1 eliminated cell debris, R2 and R3 eliminated cell doublets, R4 eliminated dead cells based on DAPI, and R5 gated CD133^{high}CD71^{low} cells. R5 was further analyzed by size (FSC-A) and granularity (SSC-A), which revealed 4 fractions. Data shown are from a representative experiment. Abbreviations: FSC-A, forward scatter-area; SSC-A, side scatter-area; FSC-W, forward scatter-width; SSC-W, side scatter-width; APC, Allophycocyanin; PE-Cy7, Phycoerythrin-cyanine dye 7.

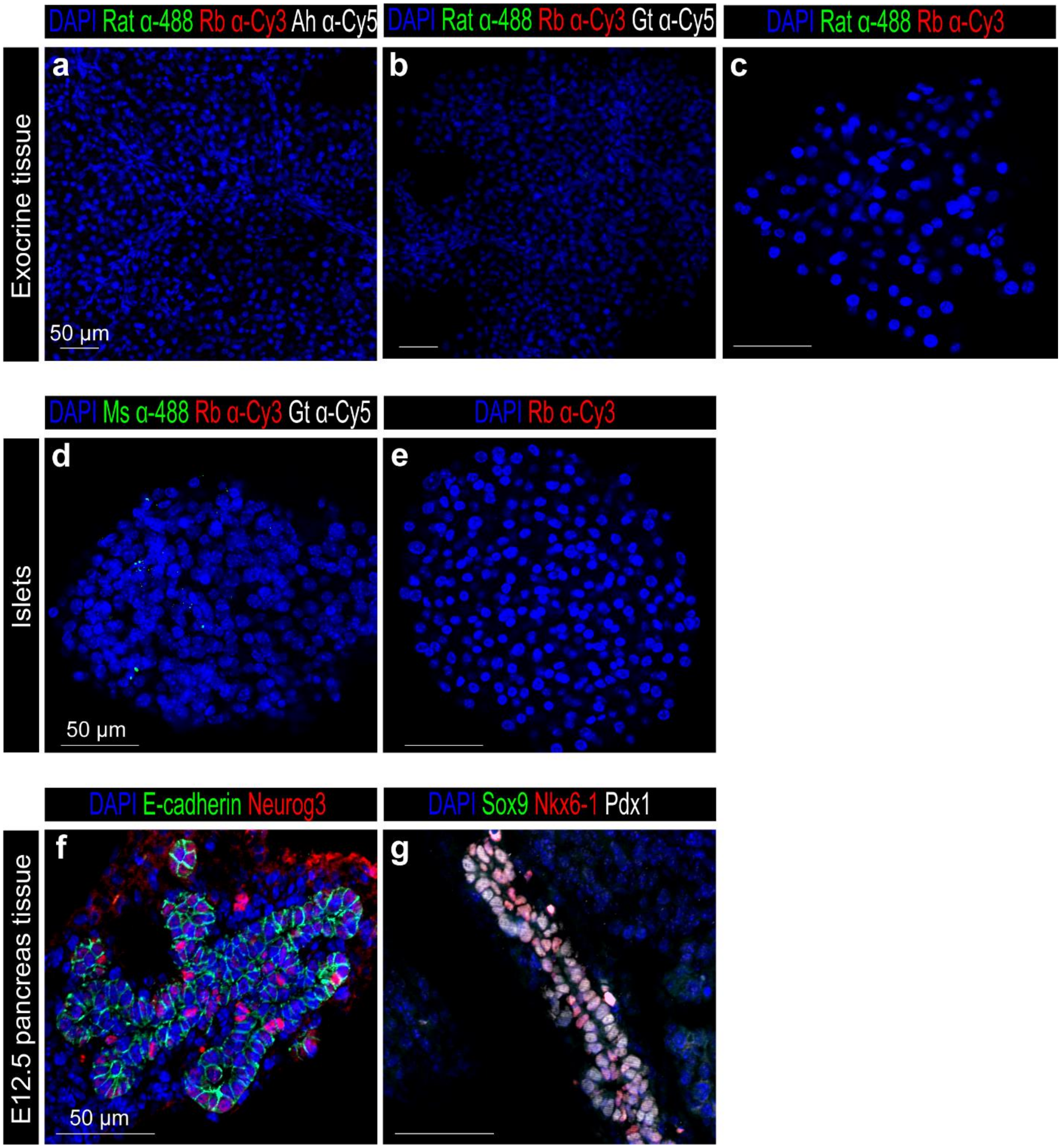

Supplementary Figure 2. Authentication of antibodies used in immunofluorescence (IF) staining with positive and negative control pancreas tissues, associated with Figure 2.

Double and triple immunofluorescence (IF) staining of adult mouse pancreatic exocrine (a-c) and islet (d-e) tissues using only secondary antibodies to confirm primary antibody specificity. (f-g) Double and triple IF staining of mouse pancreas at embryonic day 12.5 (E12.5) was used to validate the expected staining patterns of the primary antibodies that we employed. Scale bars=50 μm.

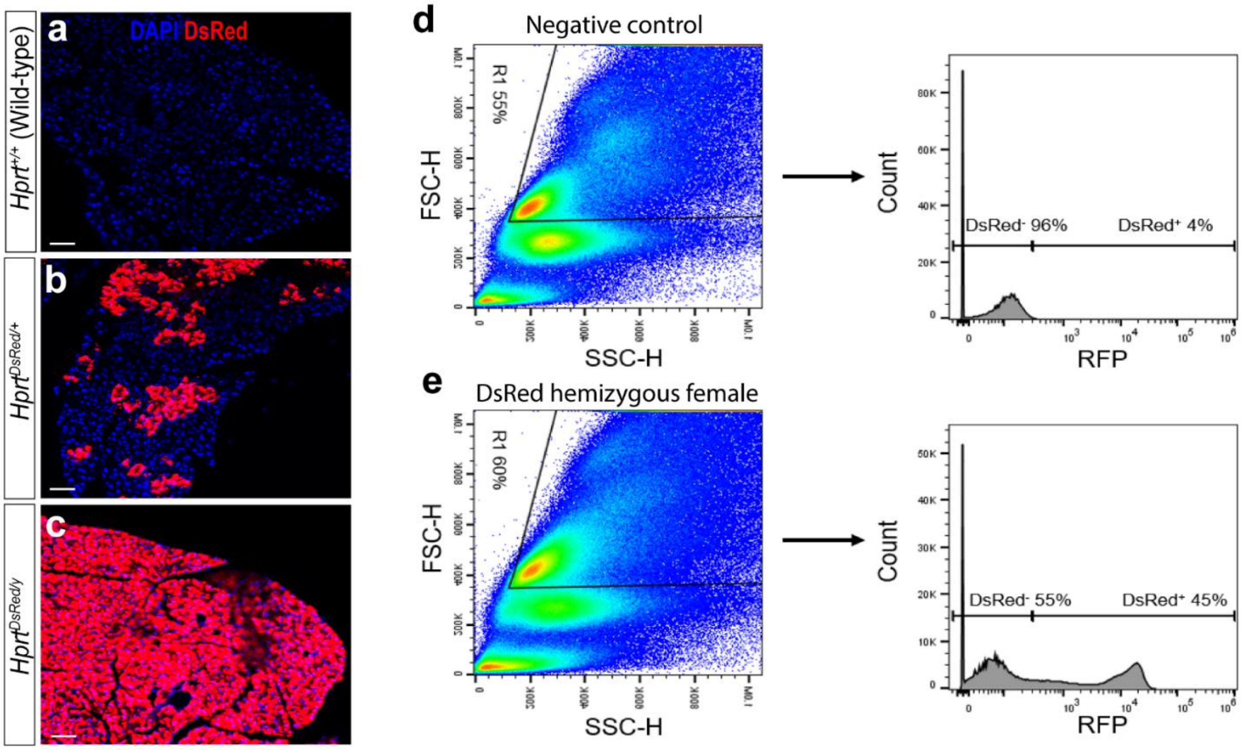

Supplementary Figure 3. Validation of random X inactivation in Hprt-dsRed heterozygous female mice, associated with Figure 4.

(a-c) Image analysis of DsRed fluorescence on pancreas from (a) wild-type female (*Hprt*$^{+/+}$) (negative control, (b) heterozygous female (*Hprt*$^{DsRed/+}$), and (c) hemizygous male (*Hprt*$^{DsRed/y}$) mice (positive control). (d-e) Splenocytes were isolated and analyzed by flow cytometry. Hprt-DsRed heterozygous female mice showed approximately half of the splenocytes expressing DsRed, consistent with a random X inactivation pattern. Scale bars=50 µm

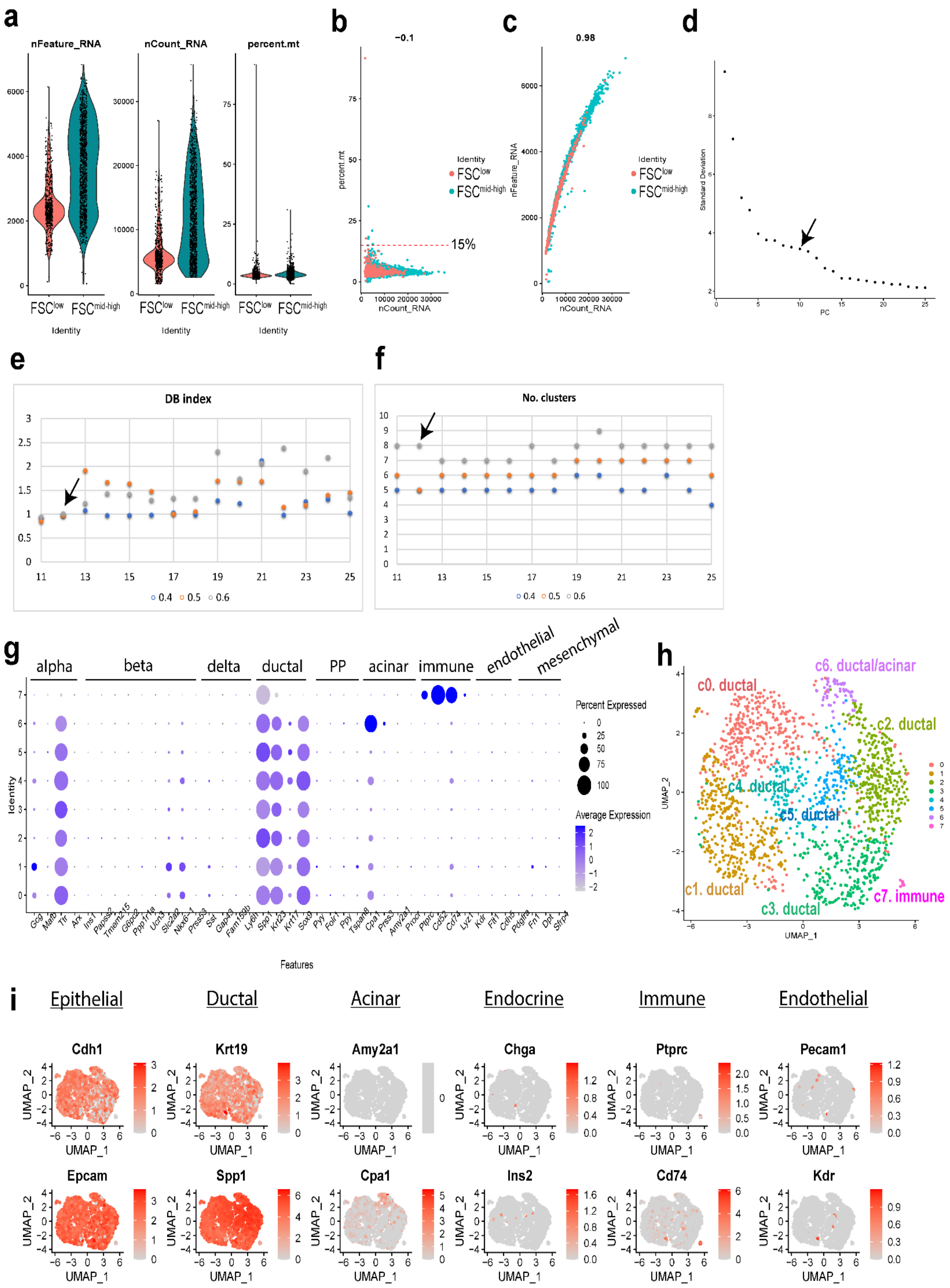
a
nFeature_RNA
nCount_RNA
percent.mt
Identity
b
-0.1
percent.mt
nCount_RNA
15%
c
0.98
nFeature_RNA
nCount_RNA
d
Standard Deviation
PC
e
DB index
f
No. clusters
g
alpha
beta
delta
ductal
PP
acinar
immune
endothelial
mesenchymal
Percent Expressed
Average Expression
Identity
Features
h
c0. ductal
c1. ductal
c2. ductal
c3. ductal
c4. ductal
c5. ductal
c6. ductal/acinar
c7. immune
UMAP_1
UMAP_2
i
Epithelial
Ductal
Acinar
Endocrine
Immune
Endothelial
Cdh1
Krt19
Amy2a1
Chga
Ptprc
Pecam1
Epcam
Spp1
Cpa1
Ins2
Cd74
Kdr

Supplementary Figure 4. Quality control of droplet-based RNA-Sequencing, cluster identification and gene expression pattern, associated with Figure 5.

$CD133^{high}CD71^{low}FSC^{low}$ and $CD133^{high}CD71^{low}FSC^{mid\text{-}high}$ fractions were subjected to droplet-based RNA-sequencing and the resulting data analyzed using the Seurat package. (a) The number of feature genes (biologically informative genes) and mRNA counts per unit showed a unimodal or bimodal distribution of events from $CD133^{high}CD71^{low}FSC^{low}$ or $CD133^{high}CD71^{low}FSC^{mid\text{-}high}$ fraction, respectively. The bimodal distribution is consistent with the presence of both single cells and small clusters. (b) Events with higher than 15% mitochondria genes were excluded. (c) The strong positive correlation (R^2 = 0.98) between the number of feature genes and the number of mRNAs per event indicates that the mRNAs identified are biologically informative. (d) The elbow plot analysis indicated that 10 principal components were sufficient to capture most of the variation in the data. (e) Davies-Bouldin (DB) index of multiple dimensions indicated that 12 dimensions contain the least variation. (f) Clustering at 12 dimensions with a 0.6x resolution results in 8 clusters (clusters 0 to 7). (g) Gene expression levels of various markers among the 8 clusters. (h) Annotated cell identify in UMAP of the combined datasets. (i) Gene expression levels of various lineage markers in the UMAP shown in (h).

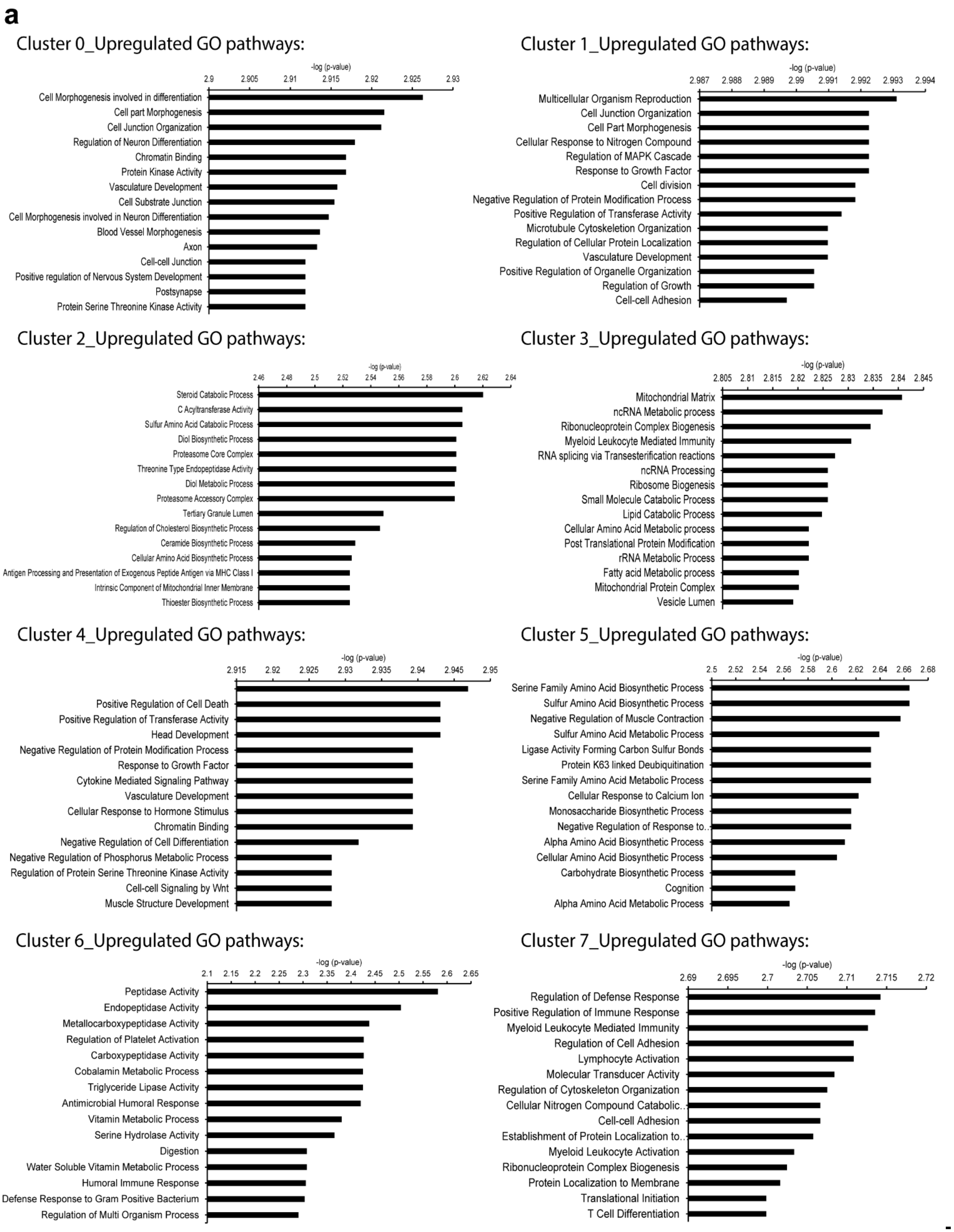
a
Cluster 0_Upregulated GO pathways:
-log (p-value)
2.9 2.905 2.91 2.915 2.92 2.925 2.93
Cell Morphogenesis involved in differentiation
Cell part Morphogenesis
Cell Junction Organization
Regulation of Neuron Differentiation
Chromatin Binding
Protein Kinase Activity
Vasculature Development
Cell Substrate Junction
Cell Morphogenesis involved in Neuron Differentiation
Blood Vessel Morphogenesis
Axon
Cell-cell Junction
Positive regulation of Nervous System Development
Postsynapse
Protein Serine Threonine Kinase Activity
Cluster 1_Upregulated GO pathways:
-log (p-value)
2.987 2.988 2.989 2.99 2.991 2.992 2.993 2.994
Multicellular Organism Reproduction
Cell Junction Organization
Cell Part Morphogenesis
Cellular Response to Nitrogen Compound
Regulation of MAPK Cascade
Response to Growth Factor
Cell division
Negative Regulation of Protein Modification Process
Positive Regulation of Transferase Activity
Microtubule Cytoskeleton Organization
Regulation of Cellular Protein Localization
Vasculature Development
Positive Regulation of Organelle Organization
Regulation of Growth
Cell-cell Adhesion
Cluster 2_Upregulated GO pathways:
-log (p-value)
2.46 2.48 2.5 2.52 2.54 2.56 2.58 2.6 2.62 2.64
Steroid Catabolic Process
C Acyltransferase Activity
Sulfur Amino Acid Catabolic Process
Diol Biosynthetic Process
Proteasome Core Complex
Threonine Type Endopeptidase Activity
Diol Metabolic Process
Proteasome Accessory Complex
Tertiary Granule Lumen
Regulation of Cholesterol Biosynthetic Process
Ceramide Biosynthetic Process
Cellular Amino Acid Biosynthetic Process
Antigen Processing and Presentation of Exogenous Peptide Antigen via MHC Class I
Intrinsic Component of Mitochondrial Inner Membrane
Thioester Biosynthetic Process
Cluster 3_Upregulated GO pathways:
-log (p-value)
2.805 2.81 2.815 2.82 2.825 2.83 2.835 2.84 2.845
Mitochondrial Matrix
ncRNA Metabolic process
Ribonucleoprotein Complex Biogenesis
Myeloid Leukocyte Mediated Immunity
RNA splicing via Transesterification reactions
ncRNA Processing
Ribosome Biogenesis
Small Molecule Catabolic Process
Lipid Catabolic Process
Cellular Amino Acid Metabolic process
Post Translational Protein Modification
rRNA Metabolic Process
Fatty acid Metabolic process
Mitochondrial Protein Complex
Vesicle Lumen
Cluster 4_Upregulated GO pathways:
-log (p-value)
2.915 2.92 2.925 2.93 2.935 2.94 2.945 2.95
Positive Regulation of Cell Death
Positive Regulation of Transferase Activity
Head Development
Negative Regulation of Protein Modification Process
Response to Growth Factor
Cytokine Mediated Signaling Pathway
Vasculature Development
Cellular Response to Hormone Stimulus
Chromatin Binding
Negative Regulation of Cell Differentiation
Negative Regulation of Phosphorus Metabolic Process
Regulation of Protein Serine Threonine Kinase Activity
Cell-cell Signaling by Wnt
Muscle Structure Development
Cluster 5_Upregulated GO pathways:
-log (p-value)
2.5 2.52 2.54 2.56 2.58 2.6 2.62 2.64 2.66 2.68
Serine Family Amino Acid Biosynthetic Process
Sulfur Amino Acid Biosynthetic Process
Negative Regulation of Muscle Contraction
Sulfur Amino Acid Metabolic Process
Ligase Activity Forming Carbon Sulfur Bonds
Protein K63 linked Deubiquitination
Serine Family Amino Acid Metabolic Process
Cellular Response to Calcium Ion
Monosaccharide Biosynthetic Process
Negative Regulation of Response to..
Alpha Amino Acid Biosynthetic Process
Cellular Amino Acid Biosynthetic Process
Carbohydrate Biosynthetic Process
Cognition
Alpha Amino Acid Metabolic Process
Cluster 6_Upregulated GO pathways:
-log (p-value)
2.1 2.15 2.2 2.25 2.3 2.35 2.4 2.45 2.5 2.55 2.6 2.65
Peptidase Activity
Endopeptidase Activity
Metallocarboxypeptidase Activity
Regulation of Platelet Activation
Carboxypeptidase Activity
Cobalamin Metabolic Process
Triglyceride Lipase Activity
Antimicrobial Humoral Response
Vitamin Metabolic Process
Serine Hydrolase Activity
Digestion
Water Soluble Vitamin Metabolic Process
Humoral Immune Response
Defense Response to Gram Positive Bacterium
Regulation of Multi Organism Process
Cluster 7_Upregulated GO pathways:
-log (p-value)
2.69 2.695 2.7 2.705 2.71 2.715 2.72
Regulation of Defense Response
Positive Regulation of Immune Response
Myeloid Leukocyte Mediated Immunity
Regulation of Cell Adhesion
Lymphocyte Activation
Molecular Transducer Activity
Regulation of Cytoskeleton Organization
Cellular Nitrogen Compound Catabolic..
Cell-cell Adhesion
Establishment of Protein Localization to..
Myeloid Leukocyte Activation
Ribonucleoprotein Complex Biogenesis
Protein Localization to Membrane
Translational Initiation
T Cell Differentiation

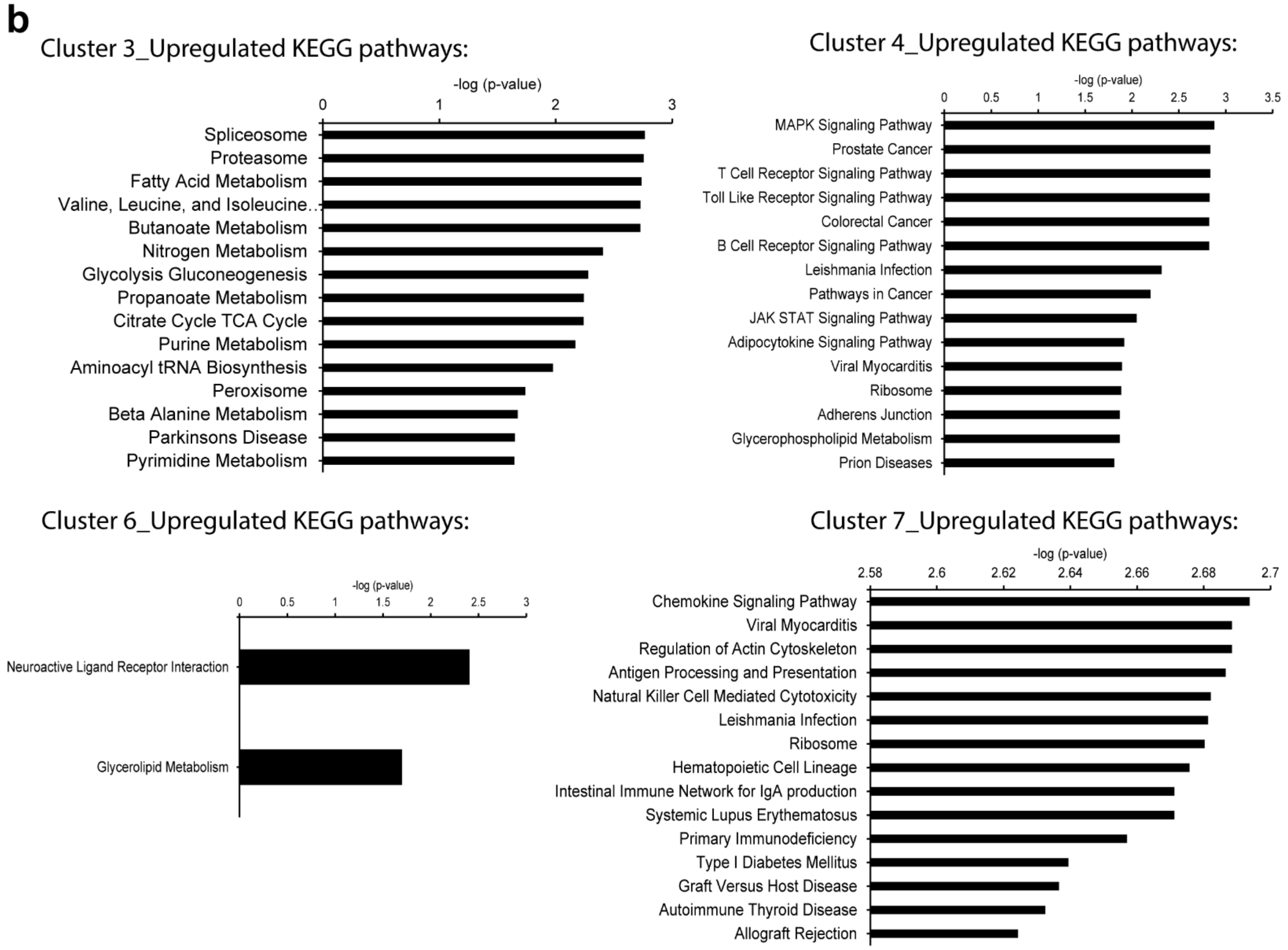

Supplementary Figure 5. Top upregulated GSEA-GO and GSEA-KEGG pathways in each cluster identified by droplet-based RNA-sequencing, associated with Figure 5.

(a) Upregulated pathways identified by Gene Set Enrichment Analysis (GSEA)-Gene Ontology (GO) for clusters 0-7. (b) Upregulated pathways identified by GSEA-Kyoto Encyclopedia of Genes and Genomes (KEGG) pathways for clusters 3, 4, 6, and 7. No significant GSEA-KEGG pathways were found for cluster 2 and 5. See also Supplementary Datasets 2-3.

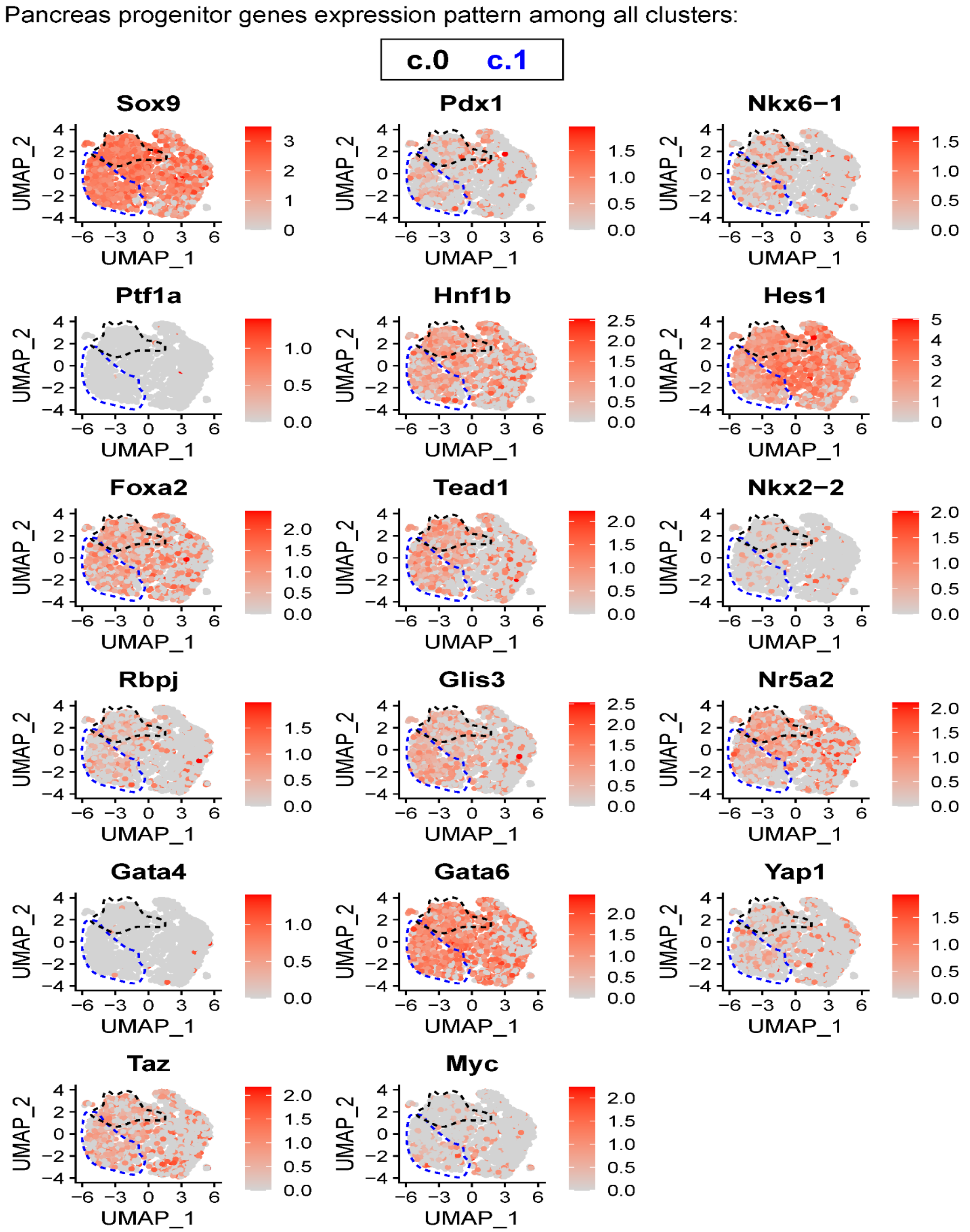

Supplementary Figure 6: Gene expression of various known pancreatic progenitor cell markers among all clusters, associated with Figure 5.

UMAPs of the combined datasets from CD133^{high}CD71^{low}FSC^{low} and CD133^{high}CD71^{low}FSC$^{mid\text{-}high}$ fractions are shown. Clusters 0 (black) and 1 (blue) are outlined in dashed lines.

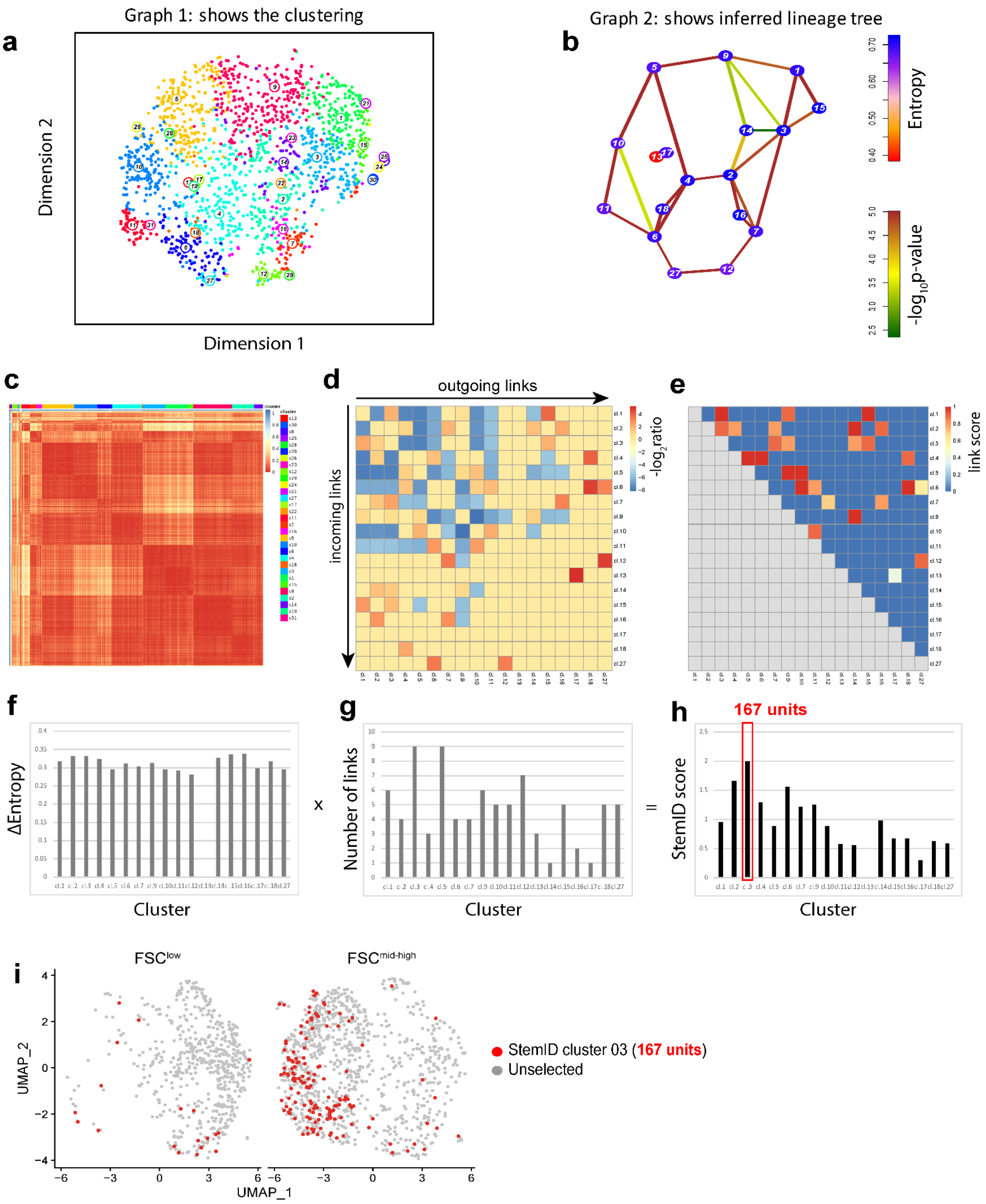

Graph 1: shows the clustering
Graph 2: shows inferred lineage tree
a
b
c
d
e
f
g
h
i
Dimension 1
Dimension 2
Entropy
$-\log_{10}$p-value
outgoing links
incoming links
$-\log_2$ratio
link score
ΔEntropy
Number of links
StemID score
Cluster
x
=
167 units
FSC^{low}
FSC$^{mid-high}$
UMAP_1
UMAP_2
StemID cluster 03 (167 units)
Unselected

Supplementary Figure 7: StemID analysis of droplet RNA-sequencing datasets, associated with Figure 5.

(a) StemID reclustered the original combined datasets. (b) Inferred lineage tree. Nodes with multiple lines indicate a higher stemness. (c) Heatmap of cell-to-cell transcriptome distances. (d) Heatmap of the incoming vs. outgoing links per cluster. (e) Heatmap of the link score per cluster. (f) Bar graph of the delta entropy (ΔEntropy) for each cluster. (g) Bar graph of the number of links for each cluster. (h) StemID score for each cluster determined by multiplying ΔEntropy with the number of links for each cluster. StemID cluster 3 showed the highest StemID score (1.99), compared to the other 17 clusters (1.66 to 0). StemID cluster 3 contained 167 units. (i) StemID cluster 3 was projected onto the original UMAP with split samples.

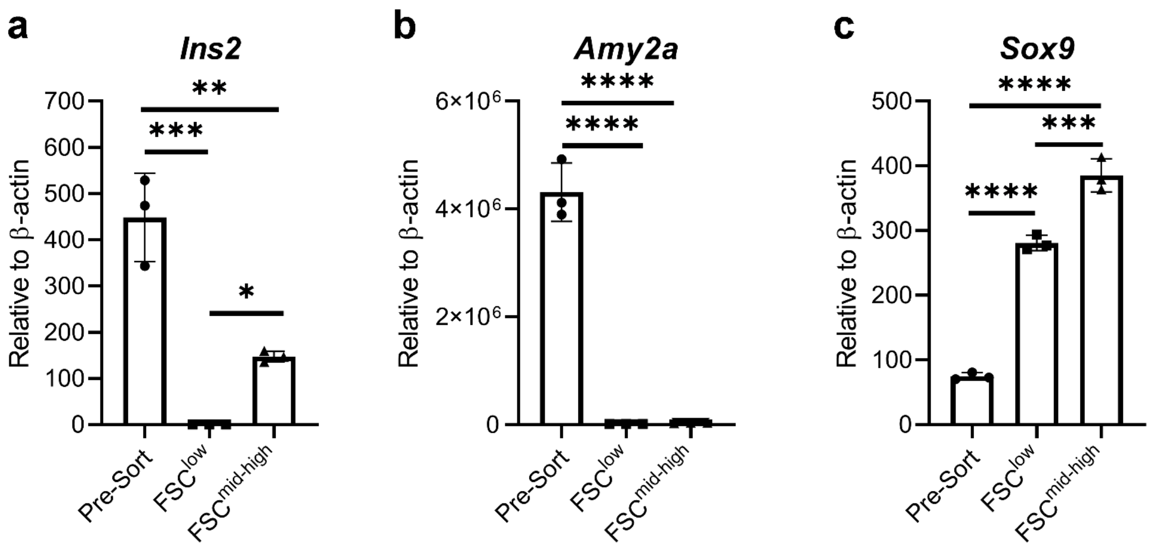

Supplementary Figure 8. Quantitative RT-PCR analysis of sorted CD133^{high}CD71^{low}FSC^{low}, CD133^{high}CD71^{low}FSC$^{mid\text{-}high}$ and pre-sort cells.

Pancreatic cells were sorted and analyzed for gene expression of *Insulin 2* (a), *Amylase2a* (b) and *Sox9* (c), using conventional qRT-PCR. Beta-actin was used as the internal control. *p<0.05, **p<0.01, ***p<0.001, ****p<0.0001, n=3. Statistics were performed using one-way ANOVA.

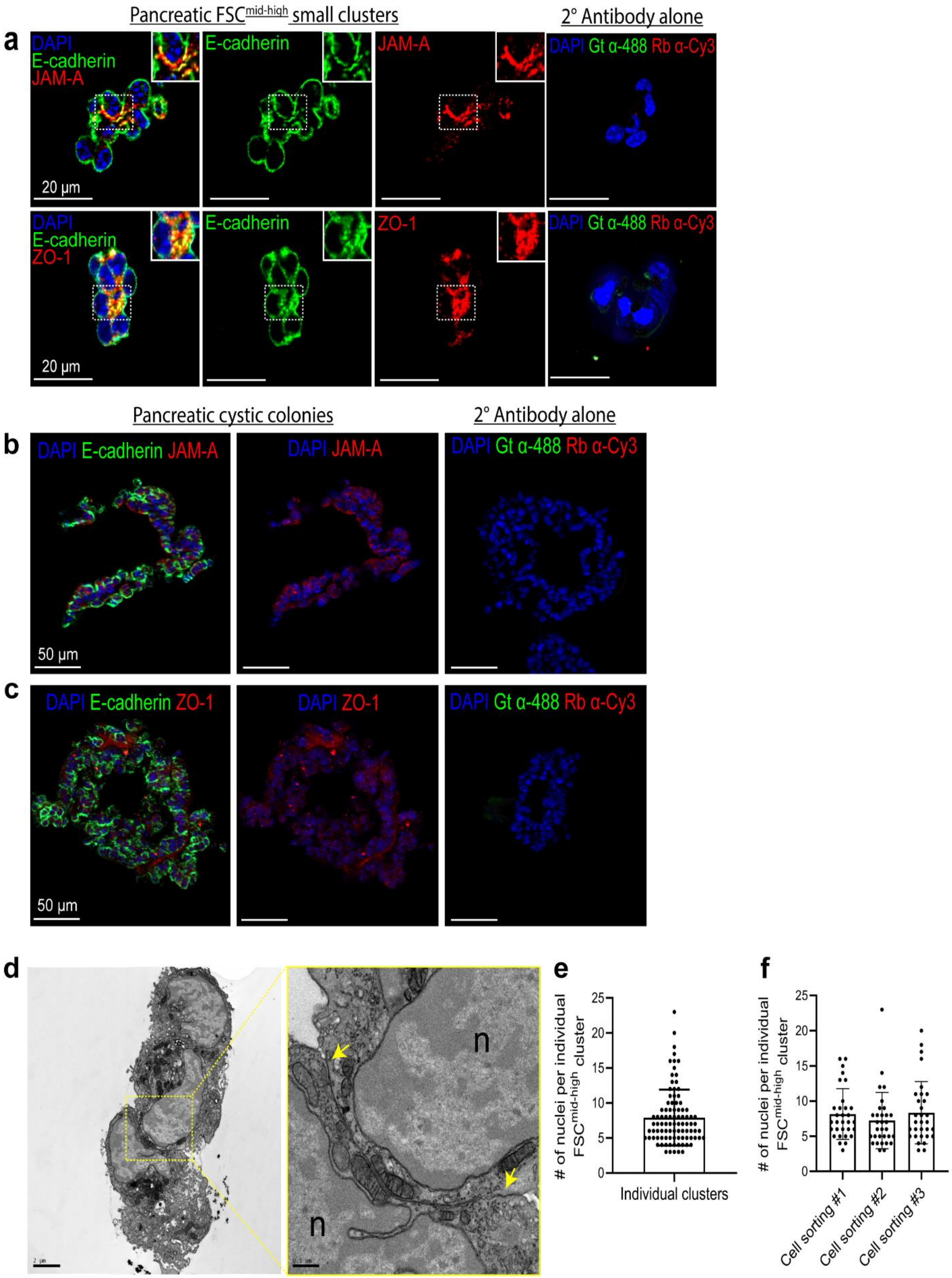

Pancreatic FSC^mid-high small clusters
2° Antibody alone
a
DAPI
E-cadherin
JAM-A
E-cadherin
JAM-A
DAPI Gt α-488 Rb α-Cy3
20 µm
DAPI
E-cadherin
ZO-1
E-cadherin
ZO-1
DAPI Gt α-488 Rb α-Cy3
20 µm
Pancreatic cystic colonies
2° Antibody alone
b
DAPI E-cadherin JAM-A
DAPI JAM-A
DAPI Gt α-488 Rb α-Cy3
50 µm
c
DAPI E-cadherin ZO-1
DAPI ZO-1
DAPI Gt α-488 Rb α-Cy3
50 µm
d
n
n
e
# of nuclei per individual FSC^mid-high cluster
Individual clusters
f
# of nuclei per individual FSC^mid-high cluster
Cell sorting #1
Cell sorting #2
Cell sorting #3

Supplementary Figure 9. Ductal small clusters express tight junction markers and contain an average of 8 cells per cluster.

(a) Co-IF staining of small clusters with tight junction marker JAMA (top) or ZO-1 (bottom) and E-cadherin (an epithelial cell marker). Small clusters treated with secondary antibodies only were used to rule out non-specific staining for E-cadherin (Goat anti-488), JAM-A and ZO-1 (Rabbit anti-Cy3). Scale bars=20 µm.
(b) Three-week-old Cystic colonies grown in Matrigel/RSPO1 colony assay expressed minimum levels of JAM-A. Panel on the right shows negative control using secondary antibodies only. DAPI (blue) identifies individual nuclei. (c) Same as (b) except that JAM-A staining was replaced with ZO-1. Scale bars=50 µm.
(d) Transmission electron microscopy (TEM) analysis of a small cluster showing cell-cell boundary between two adjacent cells, with their nuclei (n) and tight junctions (yellow arrows) indicated. Scale bar=2 or 0.5 µm, respectively. (e) Small clusters were cytospun, stained by Wright-Giemsa and the number of nuclei in each cluster was counted and presented. (f) Same as (e) but data are separated into individual experiments. Data represent mean ± SD. There were 8.1 ± 3.5 (n=31), 7.2 ± 3.9 (n=31) and 8.3 ± 4.4 (n=31) nuclei per cluster in experiments 1, 2 and 3, respectively.

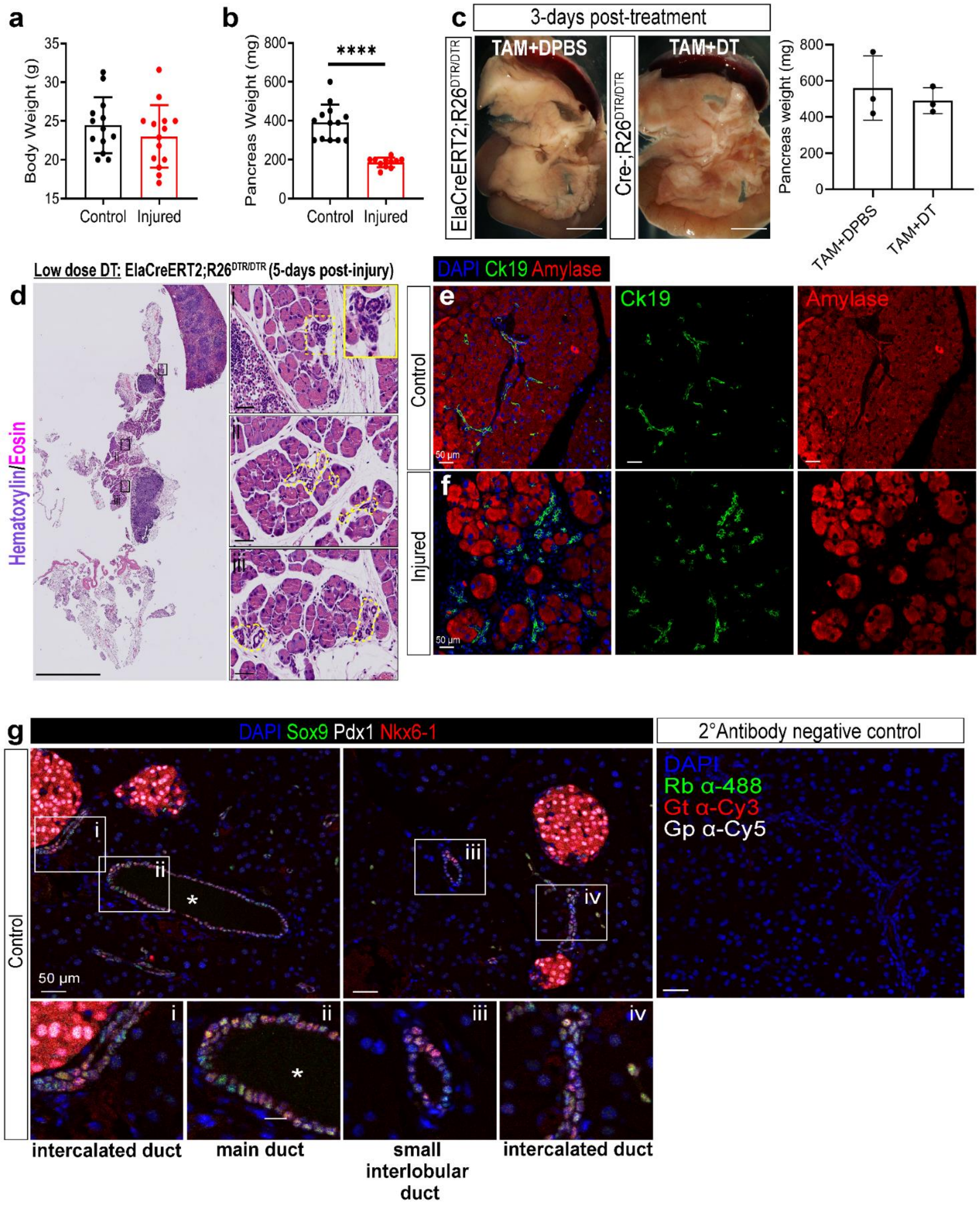

Supplementary Figure 10: High dose and low dose DT-induced acinar injury, control mice, and identification of Sox9+/Pdx1+/Nkx6.1+ triple-positive cells in normal pancreatic ducts, associated with Figure 6.

(a-b) ElaCreERT2; R26$^{DTR/DTR}$ mice were injected with TAM to induce DTR expression in the acinar cells, followed by injection with high doses of DT or vehicle control. See experimental schematic in Fig. 6c. (a) No difference in body weight between control and injured mice. (b) Pancreas weight was reduced in the acinar ablated pancreas. ****$p<0.0001$, n=13-14. (c) Examination of pancreas weight in control mice. Brightfield images of dissected pancreas, with spleen and duodenum still attached, from two types of control mice. Cre-positive control mice received TAM and vehicle DPBS. Cre-negative control mice received TAM and DT. n=3. Scale bars=2.5 mm. (d) H&E staining of an injured pancreas 5 days after the injection with low doses of DT. Boxes outlined by yellow lines highlight ductal clusters. Scale bars=2.5 mm. (e-f) Co-IF staining of a ductal marker (CK19, green), an acinar marker (Amylase, red), and DAPI (blue) in control (e) or high dose DT injured pancreas (f). Scale bars=50 µm. (g) Co-IF staining of normal adult murine pancreas with DAPI (blue), Sox9 (green), Pdx1 (white), and Nkx6-1 (red). Insets highlight different ducts: main, intercalated, and small interlobular. Negative control (secondary antibodies only) is on the right. Scale bars=50 µm.

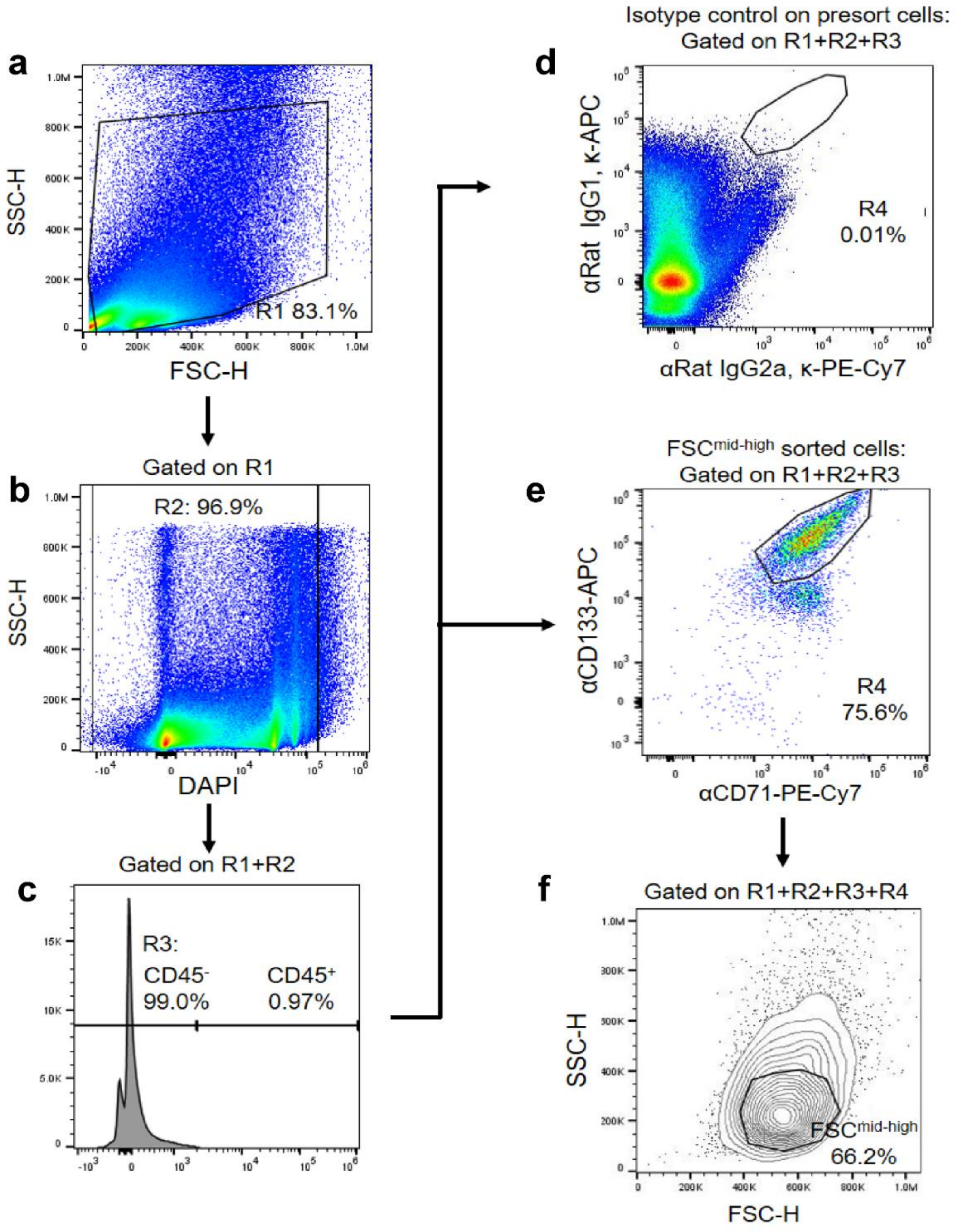

Supplementary Figure 11: Flow cytometry gating strategy, associated with Figure 6 h-i.

Due to differences in hardware between the Attune NxT and Aria-SORP cytometers, CD45^{-}CD133^{high}CD71^{low}FSC$^{mid\text{-}high}$ fraction sorted from the Aria-SORP was used to find the position of the same fraction in the Attune NxT cytometer. Briefly, on the Attune NxT cytometer, presort cells from normal mice were sequentially gated using the following regions (R). R1 eliminated cell debris (a), R2 eliminated dead cells (b), and R3 eliminated CD45^{+} cells (c). R4 identified CD45^{-}CD133^{high}CD71^{low} cells (Fig. 6h). Staining with isotype control antibodies demonstrated specificity of anti-CD133 and anti-CD71 antibodies (d). The sorted CD45^{-}CD133^{high}CD71^{low}FSC$^{mid\text{-}high}$ fraction from the Aria-SORP was confirmed to appear in the CD45^{-}CD133^{high}CD71^{low} gate (e); the events there were then further analyzed by FSC-H and SSC-H (f), which showed the position of CD45^{-}CD133^{high}CD71^{low}FSC$^{mid\text{-}high}$ fraction in the Attune NxT cytometer. This strategy was used subsequently for flow cytometry analysis shown in Fig. 6.

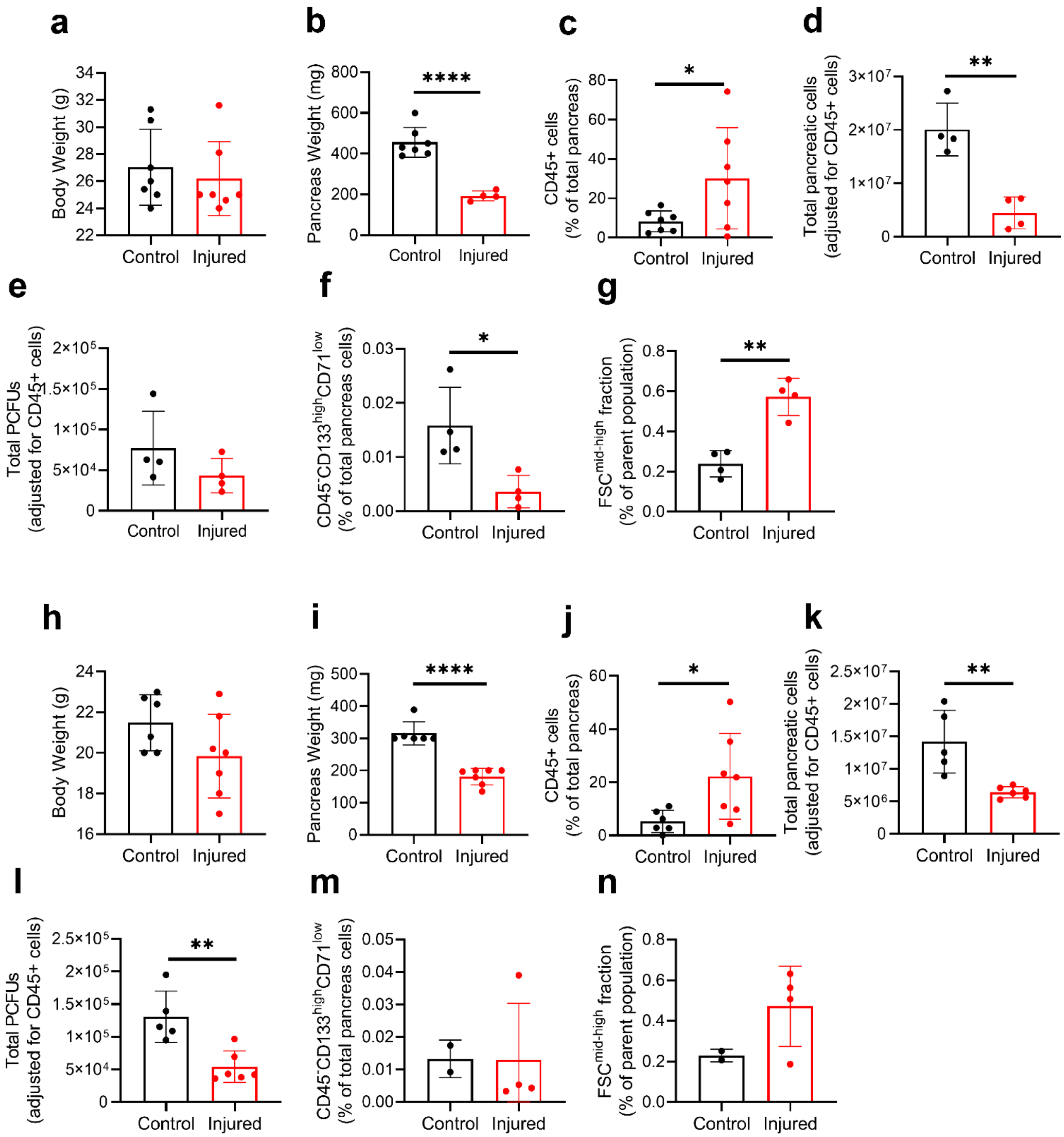

Supplementary Figure 12: Data from male and female cohorts associated with Figure 6j-n and Supplementary Figure 10a-b.

(a-g) male mice. (h-n) female mice. *p<0.05, **p<0.01, ****p<0.0001, n=2-7. Statistics were performed using two-tailed Student's t-test Welch's correction. Error bars represent SD or SEM.

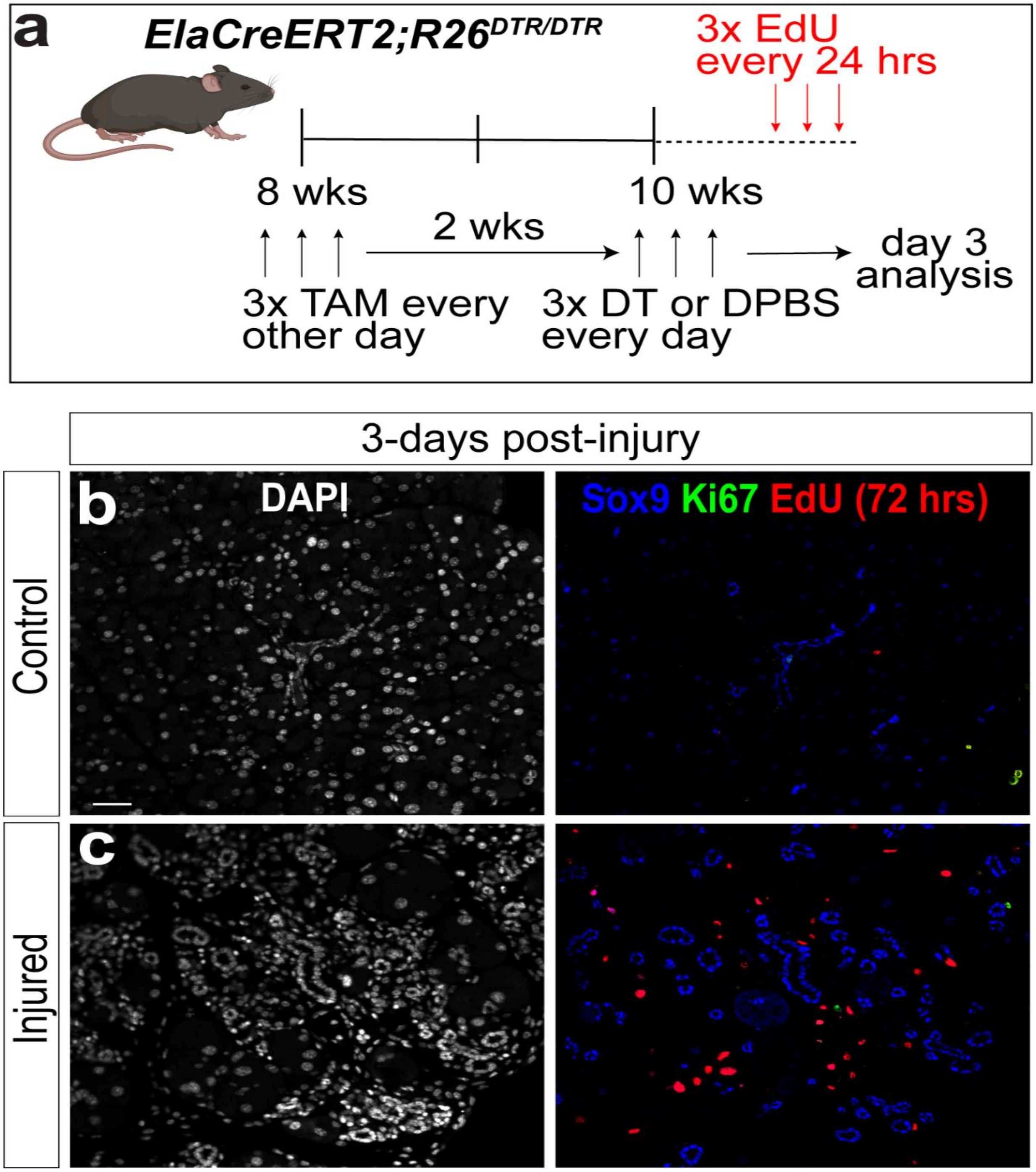

Supplementary Figure 13: Ductal clusters did not proliferate 3 days post-acinar cell injury induced with high doses of DT.

(a) Schematic of experimental design. (b-c) Proliferation analysis of control and injured pancreata 3 days after acinar injury with high doses of DT. DAPI marker (white) was used to identify individual cells. Co-IF staining of Sox9+ ductal cells (blue) with proliferation markers Ki67 (green) and EdU (red) showed that ductal cells were not proliferating. Scale bars=50 µm.

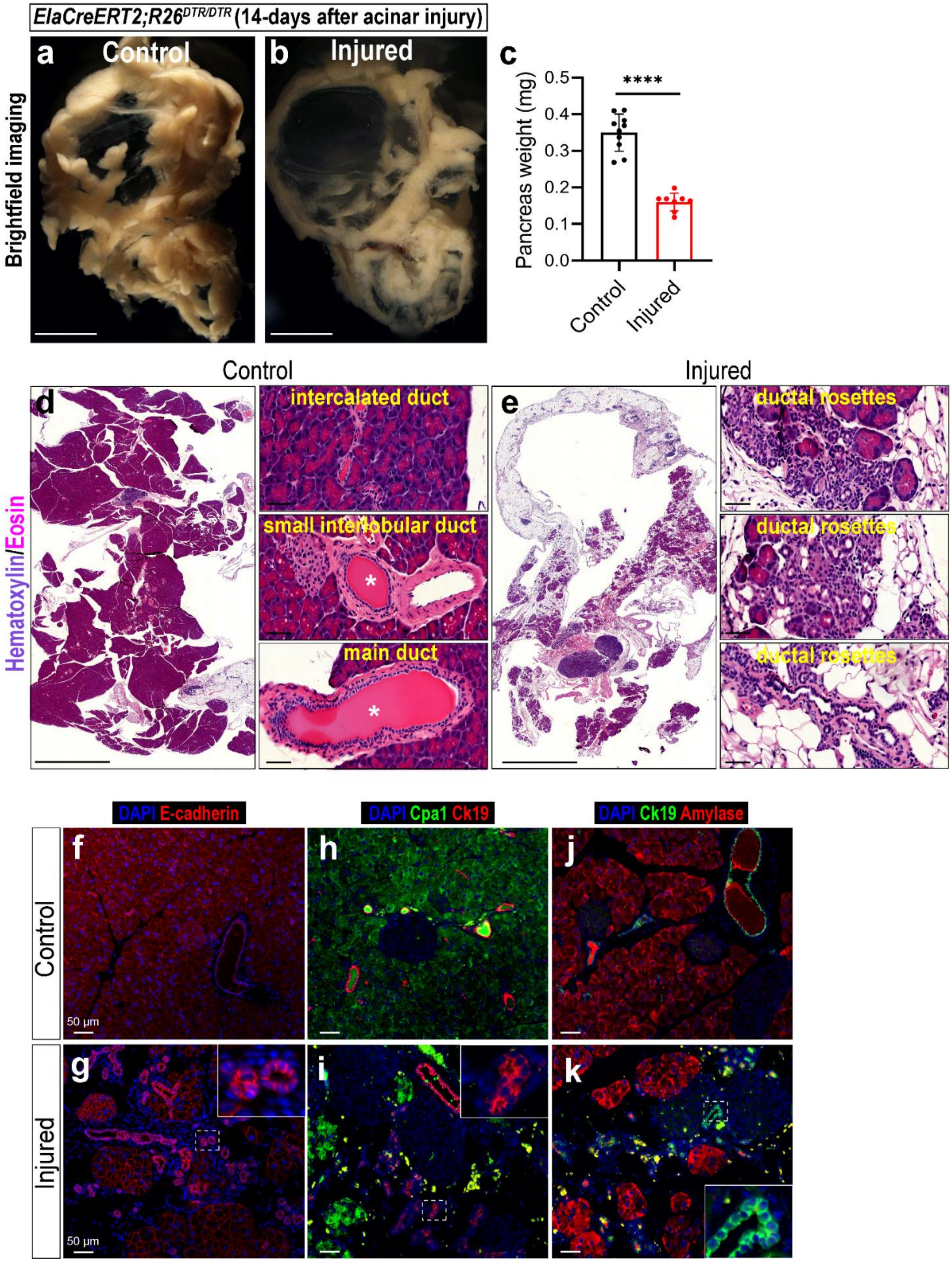
ElaCreERT2;R26DTR/DTR (14-days after acinar injury)
a Control
b Injured
c
Brightfield imaging
Pancreas weight (mg)
0.5
0.4
0.3
0.2
0.1
0.0
****
Control
Injured
Control
Injured
d
e
Hematoxylin/Eosin
intercalated duct
small interlobular duct
main duct
ductal rosettes
ductal rosettes
ductal rosettes
DAPI E-cadherin
DAPI Cpa1 Ck19
DAPI Ck19 Amylase
Control
Injured
f
g
h
i
j
k
50 µm
50 µm

Supplementary Figure 14: Ductal clusters are present 14 days post-acinar cell injury and express ductal but not acinar cell markers. (a-b) Brightfield images showing the gross morphology of pancreas from control (a) or injured mice (b) 14 days after the last DT (high dose) injection. (c) Pancreas weight was reduced in injured pancreata compared to controls. ****$p<0.0001$, n=8-10. (d-e) H&E staining of pancreas. Insets in (d) show the normal structure of intercalated, small interlobular, and main ducts in the control pancreas. White asterisk denotes lumen of small interlobular and main ducts. Insets in (e) show ductal clusters (rosettes) in injured pancreas. Scale bars=2.5 mm. (f-g) IF staining with the epithelial cell marker (E-cadherin, red) and DAPI (blue). (h-i) Co-IF staining with markers for duct (CK19, red), acinar (Cpa1, green) and nuclei (DAPI, blue). (j-k) Co-IF staining with markers for ductal (CK19, green), acinar (Amylase, red), and nuclei (DAPI, blue). Scale bars=50 µm. Cells in the ductal clusters/rosettes of injured pancreas do not simultaneously express duct and acinar markers.

Supplementary table 1. Taqman probes used for conventional and microfluidic qRT-PCR analyses.

**Murine Gene**	**Assay ID from ThermoFisher**
β-actin	Mm02619580_g1
Amy2a	Mm02342487_g1
Ca2	Mm00501572_m1
Prom1	Mm00477115_m1
Cpa1	Mm00465942_m1
Cela1	Mm00712898_m1
Gcg	Mm00801712_m1
Hnf6	Mm00447459_m1
Ins2	Mm 00731595_gH
Krt19	Mm00492980_m1
Ngn3	Mm00437606_s1
Notch2	Mm00803077_m1
Pax4	Mm01159036_m1
Ppy	Mm01250509_g1
Ucn3	Mm00453206_s1
Slc2a2	Mm 00446224_m1

Supplementary table 2. List of antibodies.

Antibodies	Dilution			Host	Company	Format or Clone name	Cat#	Lot#
	IF	WM	FACS					
C-peptide	1:100	1:800		Rabbit	Abcam	Polyclonal	ab1418	GR320539-3
Glucagon		1:1000		Mouse	Sigma	K79bB10	G2654	0000165985
Urocortin 3		1:1000		Rabbit	Phoenix Pharmaceuticals	Polyclonal	H-019-14	01293-3
Amylase	1:300	1:1000		Rabbit	Sigma	Polyclonal	A8273	061M4831
Krt19/CK19	1:500	1:500		Rabbit	Abcam	EP1580Y	ab52625	1011985-5
Krt19/CK19	1:100			Goat	Santa Cruz	Polyclonal	sc-33111	F1214
Osteopontin (Spp1)		1:1000		Goat	R&D Systems	Polyclonal	AF808	BDO0613101
Neurog3	1:100			Mouse	DSHB	Monoclonal	F25A1B3	N/A
Mucin 1	1:200	1:200		Armenian Hamster	NeoMarkers	MH1	HM-1G30-P1	1630P1810I
CPA-1	1:200			Goat	R&D Systems	Polyclonal	AF2765	WOD0122111
ZO-1	1:300			Rabbit	Thermo Fisher	ZMD.437	40-2300	UE289033
JAM-A	1:300			Rabbit	Thermo Fisher	Polyclonal	36-1700	UC283843
E-cadherin	1:300	1:100		Goat	R&D systems	Polyclonal	AF748	CYG0518081
EpCAM		1:100		Rat	DSHB	Monoclonal	G8.8	N/A
Pdx1	1:100			Guinea pig	Abcam	Polyclonal	ab47308	GR218270-1
Sox9	1:500			Rabbit	Abcam	Polyclonal	ab5535	3282152
Nkx6-1	1:100			Mouse	DSHB	Monoclonal	F55A10	
Nkx6-1	1:100			Goat	R&D Systems	Polyclonal	AF5857	CDEZ00218121
Ki67-FITC	1:200			Rat	Invitrogen	SolA15	11-5698-82	2040334
CD133	1:100			Rat	Millipore Sigma	13A4	MAB4310	2855593
CD133-Biotin			1:100	Rat	Invitrogen	13A4	13-1331-82	2002734
CD71-PE-Cy7			1:50	Rat	BioLegend	R17217	113812	B259648
CD45-FITC			1:100	Rat	Thermo Fisher	30-F11	11-0451-82	2015766
Streptavidin-APC			1:100	N/A	BioLegend	N/A	405207	B288873
Ultra-LEAF purified CD16/32			1:10	Rat	BioLegend	93	101330	B287426
**Cy™3 AffiniPure F(ab')$_2$ Fragment Donkey Anti-Rabbit IgG (H+L)**	1:2000 (frozen)<br>1:500 (paraffin)	1:2000		Donkey	Jackson ImmunoResearch		711-166-152	

**Alexa Fluor® 488 AffiniPure Anti-Rabbit IgG (H+L)**	1:1000 (frozen)<br>1:500 (paraffin)	1:1000		Donkey	Jackson ImmunoResearch		711-546-152	
**Cy™3 AffiniPure Anti-Goat IgG (H+L)**	1:2000 (frozen)<br>1:500 (paraffin)	1:2000		Donkey	Jackson ImmunoResearch		705-166-147	
**Alexa Fluor® 488 AffiniPure Anti-Goat IgG (H+L)**	1:1000 (frozen)<br>1:500 (paraffin)	1:1000		Donkey	Jackson ImmunoResearch		705-545-147	
**Alexa Fluor® 488 AffiniPure Anti-Guinea Pig IgG (H+L)**	1:1000 (frozen)<br>1:500 (paraffin)	1:1000		Donkey	Jackson ImmunoResearch		706-546-148	
**Alexa Fluor® 647 AffiniPure Anti-Guinea Pig IgG (H+L)**	1:500 (frozen)<br>1:500 (paraffin)	1:500		Donkey	Jackson ImmunoResearch		706-606-148	
**Cy™3 AffiniPure Anti-Mouse IgG (H+L)**	1:2000 (frozen)<br>1:500 (paraffin)	1:2000		Donkey	Jackson ImmunoResearch		715-166-150	
**Alexa Fluor® 488 AffiniPure Anti-Rat IgG (H+L)**	1:1000 (frozen)<br>1:500 (paraffin)	1:1000		Donkey	Jackson ImmunoResearch		712-545-153	
**Alexa Fluor® 488 AffiniPure Anti-Armenian Hamster IgG (H+L)**	1:1000 (frozen)<br>1:500 (paraffin)	1:1000		Goat	Jackson ImmunoResearch		127-545-160	
**4,6-diamidino-2-phenylindole (DAPI)**	1:2000 (frozen)<br>1:1000 (paraffin)	1:2000		N/A	Thermo Fisher Scientific		D1306	

Reference

1 Criscimanna A, Speicher JA, Houshmand G et al. Duct cells contribute to regeneration of endocrine and acinar cells following pancreatic damage in adult mice. Gastroenterology 2011;141(4):1451-1462, 1462 e1451-1456.

2 Bevis BJ, Glick BS. Rapidly maturing variants of the Discosoma red fluorescent protein (DsRed). Nat Biotechnol 2002;20(1):83-87.

3 Tang SH, Silva FJ, Tsark WM et al. A Cre/loxP-deleter transgenic line in mouse strain 129S1/SvImJ. Genesis 2002;32(3):199-202.

4 Jin L, Gao D, Feng T et al. Cells with surface expression of CD133 high CD71 low are enriched for tripotent colony-forming progenitor cells in adult murine pancreas. Stem Cell Res 2016;16(1):40-53.

5 Jin L, Feng T, Shih HP et al. Colony-forming cells in the adult mouse pancreas are expandable in Matrigel and form endocrine/acinar colonies in laminin hydrogel. Proc Natl Acad Sci U S A 2013;110(10):3907-3912.

6 Winkler M, Trieu N, Feng T et al. A quantitative assay for insulin-expressing colony-forming progenitors [in eng]. J Vis Exp 2011(57):e3148.

7 Tremblay JR, LeBon JM, Luo A et al. In Vitro Colony Assays for Characterizing Tri-potent Progenitor Cells Isolated from the Adult Murine Pancreas. J Vis Exp 2016(112).

8 Bankhead P, Loughrey MB, Fernandez JA et al. QuPath: Open source software for digital pathology image analysis. Sci Rep 2017;7(1):16878.
